# *Sphenomorphus tamchucensis* sp. nov. (Squamata, Scincidae), a new skink from Vietnam

**DOI:** 10.3897/zookeys.1266.176724

**Published:** 2026-01-13

**Authors:** Anh Van Pham, Cuong The Pham, Linh Thuy Ha, Minh Duc Le, Tien Quang Phan, Anh Ngoc Thi Ho, Minh Le, Truong Quang Nguyen

**Affiliations:** 1 Faculty of Environmental Sciences, University of Science, Vietnam National University, Hanoi, 334 Nguyen Trai Road, Hanoi 11416, Vietnam University of Science, Vietnam National University, Hanoi Hanoi Vietnam; 2 Institute of Biology, Vietnam Academy of Science and Technology, 18 Hoang Quoc Viet Road, Hanoi 10072, Vietnam Institute of Biology, Vietnam Academy of Science and Technology Hanoi Vietnam; 3 Graduate University of Science and Technology, Vietnam Academy of Science and Technology, 18 Hoang Quoc Viet Road, Hanoi 10072, Vietnam Graduate University of Science and Technology Hanoi Vietnam; 4 Central Institute for Natural Resources and Environmental Studies, Vietnam National University, Hanoi, 19 Le Thanh Tong, Hanoi 11021, Vietnam Vietnam National University, Hanoi Hanoi Vietnam; 5 Department of Herpetology, American Museum of Natural History, Central Park West at 79th Street, New York 10024, USA Department of Herpetology, American Museum of Natural History New York United States of America

**Keywords:** COI, Kim Bang, molecular phylogeny, morphology, Ninh Binh Province, taxonomy

## Abstract

A new skink species, *Sphenomorphus tamchucensis***sp. nov**., is described from Ninh Binh Province, northern Vietnam, based on morphological and molecular evidence. The new species can be distinguished from other *Sphenomorphus* species by a combination of the following characteristics: size medium (maximal SVL 41.5 mm); primary temporals two; external ear opening without lobules; loreals two; supralabials seven; infralabials six; nuchals absent; midbody scales in 28 rows; dorsal scales smooth, in six rows across the back; paravertebral scales 58–63, not widened; ventral scales in 55–61 rows; 8–10 smooth lamellae beneath finger IV and 13–15 beneath toe IV; toes not reaching to fingers when limbs adpressed along body; dorsal surface of body and tail bronze brown with many tiny dark dots and a discontinuous dark vertebral stripe, from middle of neck to tail base; a black stripe, in two scales wide, running from nostril to eye and extending from posterior margin of eye along upper part of flank to middle of tail. In phylogenetic analyses, the new species is recovered as an independent lineage with no clear sister taxon and at least 17.85% genetic divergence from other congeners based on a fragment of the mitochondrial COI gene.

## Introduction

The genus *Sphenomorphus* Fitzinger, 1843 currently contains 118 recognized species. In Vietnam, 17 species of the genus have been documented, including new country records and new species discoveries (Uetz et al. 2025). Nguyen et al. (2011) described *Sphenomorphus tonkinensis* Nguyen et al., 2011 from Hai Phong City, in northern Vietnam. Four new species have been discovered since 2013 in southern Vietnam, namely *S. phuquocensis* Grismer et al., 2020 from Phu Quoc Island, *S. sheai* Nguyen et al., 2013 from Kon Tum Plateau, and *S. valentinae* Bragin et al., 2025 and *S. yersini* Nguyen et al., 2018 from Khanh Hoa Province (Nguyen et al. 2013; Nguyen et al. 2018; Grismer et al. 2020; Bragin et al. 2025). In addition, Grismer et al. (2019) and Nguyen et al. (2011) transferred *Lygosoma annamiticum* Boettger, 1901, *Livorimica bacboensis* Eremchenko, 2003 and *Leptoseps tetradactylus* Darevsky & Orlov, 2005 to the genus *Sphenomorphus* Fitzinger, 1843. Recently, Bragin et al. (2025) re-assigned *Lygosoma veunsaiense* Geissler, Hartmann & Neang, 2012 to the genus *Sphenomorphus* and considered *S. sheai* is a junior synonym of *S. veunsaiense*.

During our fieldwork in karst forests of Ninh Binh Province, northern Vietnam, several skinks were captured in the Tam Chuc forest within the proposed Kim Bang Species and Habitat Conservation Area (SHCA) (Suppl. material 1). The collected specimens were assigned to the genus *Sphenomorphus* based on molecular and morphological data. Further morphological and molecular analyses showed that they are distinctly differentiated from all other existing species. We therefore describe this population of *Sphenomorphus* from Ninh Binh Province as a new species.

## Materials and methods

### Sampling

A field survey was conducted in April 2025 in Kim Bang SHCA, Ninh Binh Province, northern Vietnam (Suppl. material 1). Skinks were collected by hand between 18:30 and 22:00. After having been photographed in life, skinks were anesthetized and euthanized in a closed vessel with a piece of cotton wool containing ethyl acetate (Simmons 2002), fixed in 85% ethanol for 10 hours, and transferred to 75% ethanol for permanent storage. Tissue samples were separately preserved in 70% ethanol before fixation. Voucher specimens were deposited in the collections of the Institute of Biology (**IB**), Vietnam Academy of Science and Technology.

### Molecular data and phylogenetic analyses

We sequenced two samples of the new population from Ninh Binh Province. Additionally, 12 ingroup and one outgroup taxa were included in the phylogenetic analyses following Nguyen et al. (2018) (Suppl. material 2: table S1). Tissue samples were extracted using DNeasy blood and tissue kit, Qiagen (Hilden, Germany). Extracted DNA from the fresh tissue was amplified by DreamTaq PCR mastermix (Thermo Fisher Scientific, Lithuania). A fragment of the mitochondrial cytochrome c oxidase subunit I (COI) gene was sequenced using the primer pair LCO1490 (5'-GGT CAA CAA ATC ATA AAG ATA TTG G- 3') and HCO2198 (5'-TAA ACT TCA GGG TGA CCA AAA AAT CA-3') (Folmer et al. 1994; Jeong et al. 2013). The PCR volume consisted of 19 μl (10 μl of mastermix, 5 μl of water, 2 μl of each primer at 10 pmol/μl, and 2 μl of DNA or higher depending on the quantity of DNA in the final extraction solution). PCR condition was 95 °C for 5 min to activate the taq, with 40 cycles at 95 °C for 30 s, 50 °C for 45 s, 72 °C for 60 s, and the final extension at 72 °C for 6 min. PCR products were subjected to electrophoresis through a 1% agarose gel, 1^st^ BASE (Selangor, Malaysia). Gels were stained for 10 min in 1× TBE buffer at 2 pg/ml of ethidium bromide and visualized under UV light. Successful amplifications were purified to eliminate PCR components using GeneJET™ PCR Purification Kit (Thermo Fisher Scientific, Lithuania). Clean PCR products were sent to 1^st^ Base (Malaysia) for sequencing. Sequences generated in this study were edited using Geneious v. 7.1.8 (Kearse et al. 2012).

After sequences were aligned by Clustal X v. 2 (Thompson et al. 1997), data were analyzed using maximum parsimony (MP), as implemented in PAUP*4.0b10 (Swofford 2001), and Bayesian inference (BI), as implemented in MrBayes v. 3.2.7 (Ronquist et al. 2012). For MP analysis, heuristic analysis was conducted with 100 random taxon addition replicates using tree-bisection and reconnection (TBR) branch swapping algorithm, with no upper limit set for the maximum number of trees saved. Bootstrap support was calculated using 1000 pseudo-replicates and 100 random taxon addition replicates. All characters were equally weighted and unordered. For the maximum-likelihood (ML) analysis, we used IQ-TREE v. 2.3.4 (Nguyen et al. 2015) with a single model and 10,000 ultrafast bootstrap replications. The optimal model for nucleotide evolution was determined using jModeltest v. 2.1.4 (Darriba et al. 2012).

For Bayesian analyses, we used the optimal model selected by jModeltest with parameters estimated by MrBayes v. 3.2.7. Two independent analyses with four Markov chains (one cold and three heated) were run simultaneously for ten million generations with a random starting tree and sampled every 1000 generations. Log-likelihood scores of sample points were plotted against generation time to determine stationarity of Markov chains. Trees generated before log-likelihood scores reached stationarity were discarded from the final analyses using the burn-in function. The posterior probability values for all clades in the final majority rule consensus tree were provided. The optimal model for nucleotide evolution was set to TIM2+I+G for ML and single-model Bayesian analyses as selected by jModeltest v. 2.1.4. The cutoff point for the burn-in function was set to 25% of trees generated. Nodal support was also evaluated using bootstrap replication (BP) as estimated in PAUP, ultrafast bootstrap (UFB) in IQ-TREE v. 2.3.4, and posterior probabilities (PP) in MrBayes v. 3.2.7. BP ≥ 70, PP ≥ 0.95, and UBP ≥ 95 were regarded as strong support for a clade (Hillis and Bull 1993; Ronquist et al. 2012; Nguyen et al. 2015). Uncorrected pairwise divergences were calculated in PAUP*4.0b10.

### Morphological examination

Measurements were taken with digital calipers to the nearest 0.1 mm. The following morphological characteristics were recorded (after Nguyen et al. 2011):

**SVL** snout–vent length (from tip of snout to cloaca)

**TaL** tail length (from cloaca to tip of tail)

**AG** distance from posterior junction of forelimb and body wall to anterior junction of hindlimb and body wall (with the limbs held at right angles to the body)

**HL** head length (from tip of snout to posterior margin of parietal or interparietal, depending on the longest distance)

**HW** head width (at the widest portion of temporal region)

**HH** head height (at the deepest portion of temporal region)

**SL** snout length (from anterior margin of eye to tip of snout)

**STL** distance from snout to anterior border of tympanum

**SFlL** snout-forelimb length (from tip of snout to anterior junction of forelimb and body wall to the tip of fourth finger, with the limb held at right angles to the body)

**END** distance from anterior margin of eye to posterior border of nostril

**EL** eye length (distance between anterior and posterior corners of eyelid)

**WIN** window length (distance between anterior and posterior corners of window)

**TYD** maximum diameter of tympanum

**FlL** forelimb length (from anterior junction of forelimb and body wall to the tip of fourth finger, with the limb held at right angles to the body)

**HlL** hindlimb length (anterior junction of hindlimb and body wall to the tip of fourth finger, with the limb held at right angles to the body)

### Scalation

NSB: number of scales bordering posterolateral border of parietal; So: Supraoculars; Nu: nuchals; Lo: loreals; PC: preoculars; PS: presuboculars; SC: supraciliaries; Po: postoculars; Pos: postsuboculars; PT: primary temporals; ST: secondary temporals; SL: supralabials; AL: auricular lobules; IF: infralabials; CS: chin shields (pairs); MB: midbody scale rows; PV: paravertebral scale rows; VR: ventral scale rows; PR: precloacals; FL4: subdigital lamellae on fourth finger; TL4: subdigital lamellae on fourth toe. Bilateral scale counts were given as left/right. Sex identification was performed by inspection of presence or absence of hemipenes.

### Statistical analyses

For the statistical analyses, the newly discovered population was compared to their closest relatives based on the phylogeny of *Sphenomorphus*. Raw morphological data used for the analyses were obtained from the new specimens collected in Kim Bang SHCA, the specimens of *Sphenomorphus indicus* (Gray, 1853), collected in Son La Province, and from 52 specimens representing the five other *Sphenomorphus* species, available from previous studies (Nguyen 2011; Nguyen et al. 2011).

All statistical analyses were conducted in R v. 4.5.2 (R Core Team 2025). To remove the effects of allometry in the morphometric characters, morphometric data were also normalized to adjust raw data of morphometrics using the following equation:

*X*adj = log(*X*) − ß[log(SVL) − log(SVLmean)]

where:

*X*adj = adjusted value;

*X* = measured value;

ß = unstandardized regression coefficient for each sample;

SVLmean = overall average SVL of all samples (Thorpe 1975, 1983; Turan 1999; Lleonart et al. 2000; Chan and Grismer 2025).

A Levene’s test was used to assess homogeneity of variances for both meristic and transformed morphometric characters. As all characters showed equal variances (*p* ≥ 0.05), a one-way analysis of variance (ANOVA) followed by Tukey’s HSD post hoc test was applied to identify significant pairwise differences between species pairs.

Morphospatial relationships among species were visualized using principal component analysis (PCA) based on 13 size-corrected morphometric characters (SVL, AG, SL, STL, SFlL, END, EL, HL, HW, HH, TYD, FlL, HlL) and 13 meristic characters (So, Nu, PC, PS, SC, PT, SL, IF, MB, PV, VR, FL4, TL4). PCA was conducted using the. PCA was conducted using the ADEGENET package in R (Jombart et al. 2010) to assess whether the distribution of individuals in morphospace aligned with the putative species boundaries inferred from molecular phylogenetic analyses and supported by univariate statistics. The PCA, implemented via the “prcomp()” function in R, is an unsupervised method that captures overall variance among populations without assuming predefined groupings.

## Results

### Phylogenetic analyses

**Figure 1.**
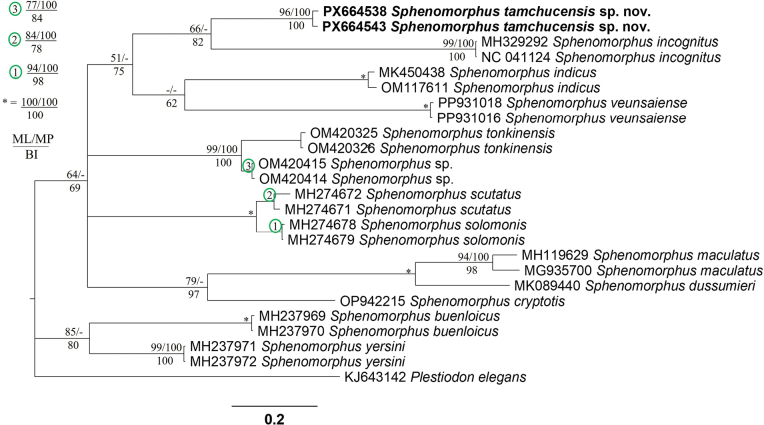
Phylogram based on the Bayesian analysis. Numbers above and below branches are ML ultrafast bootstrap/MP bootstrap values and single-model Bayesian posterior probabilities (> 50%), respectively. Dash indicates values < 50%. Letters and numbers before species names are GenBank accession records for ingroup and outgroup taxa retrieved from GenBank and specimen vouchers of the new species.

The aligned matrix of the molecular data contained 650 characters with no gaps, of which 242 were parsimony informative. The MP analysis produced two most parsimonious trees (tree length = 1053, consistency index = 0.42, retention index = 0.67). All analyses generated tree topologies with weak support along their spine, rendering the basal nodes unresolved. The new species from Ninh Binh Province was weakly recovered as the sister taxon to a clade consisting of *Sphenomorphus incognitus* (Thompson, 1912) (BP < 50, UBP = 66, PP = 82) (Fig. 1). *Sphenomorphus incognitus* is distributed in both northern Vietnam and China. In terms of genetic divergence, the new species is separated from *S. incognitus* by 19.23–19.69%. It differs from the remaining species of *Sphenomorphus* included in the study by at least ~17.85% from *S. solomonis* (Boulenger, 1887) in uncorrected pairwise genetic distance based on a fragment of the mitochondrial COI gene (Suppl. material 2: table S2).

### Statistical analyses

**Figure 2.**
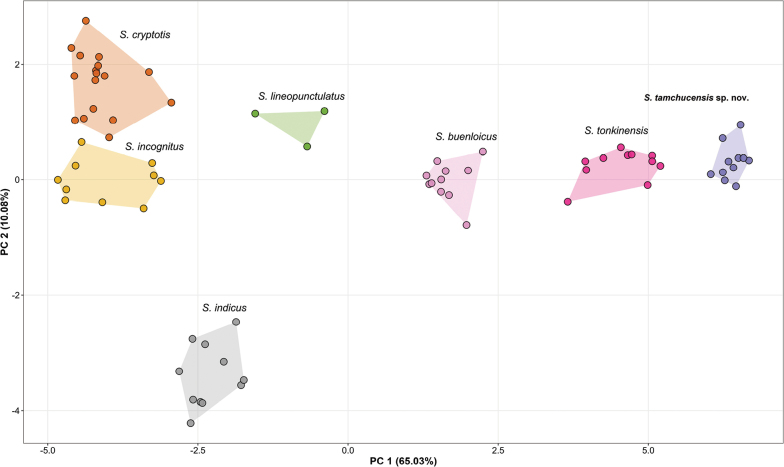
Principal component analysis (PCA) of *Sphenomorphus* species showing their morphospatial relationships along the first two principal components based on the meristic and size-corrected morphometric characters.

The ANOVA and TukeyHSD post hoc tests of the transformed morphometric and meristic characters show that the *Sphenomorphus* population from Kim Bang SHCA is significantly different from *S. buenloicus* Darevsky & Nguyen, 1983 in all size-corrected morphometric characters and four meristic characters (PC, PT, MB, FL4, TL4); from *S. cryptotis* Darevsky, Orlov & Ho, 2004 in all size-corrected morphometric characters and 11 meristic characters (So, Nu, PC, PS, SC, PT, MB, PV, VR, FL4, TL4); from *S. incognitus* in all size-corrected morphometric characters and eight meristic characters (PC, SC, IF, MB, PV, VR, FL4, TL4); from *S. indicus* in all size-corrected morphometric characters and ten meristic characters (Nu, PS, SC, PT, IF, MB, PV, VR, FL4, TL4); from *S. lineopunctulatus* Taylor, 1962 in 12 size-corrected morphometric characters (Except for SVL) and seven meristic characters (PS, SC, MB, PV, VR, FL4, TL4); and from *S. tonkinensis* in six size-corrected morphometric characters (AG, SL, END, EL, FlL, HlL) and seven meristic characters (PS, SC, MB, PV, VR, FL4, TL4) (Suppl. material 2: tables S3, S4). In the PCA analysis (Suppl. material 2: tables S5–S7), the first four extracted principal components PC1, PC2, PC3, and PC4 explain 65.0%, 10.1%, 5.8%, and 5.3% of the total variance, respectively, cumulatively accounting for 86.2%. The clustering pattern in the PCA scatterplot (Fig. 2) based on normalized morphometric and meristic data shows that PC1 and PC2 together account for 75.11% of the variation. The samples from Kim Bang clearly form distinct clusters that do not overlap with those of other species along the ordination of the first two components.

### Taxonomic account

#### Sphenomorphus tamchucensis sp. nov.

Taxon classificationAnimaliaSquamataScincidae

1DC5EA8F-9A6A-55F6-8795-00D2F993B350

https://zoobank.org/7EC0AB36-46BC-4A37-894D-2411FA9EA976

##### Material examined.

**Figure 3.**
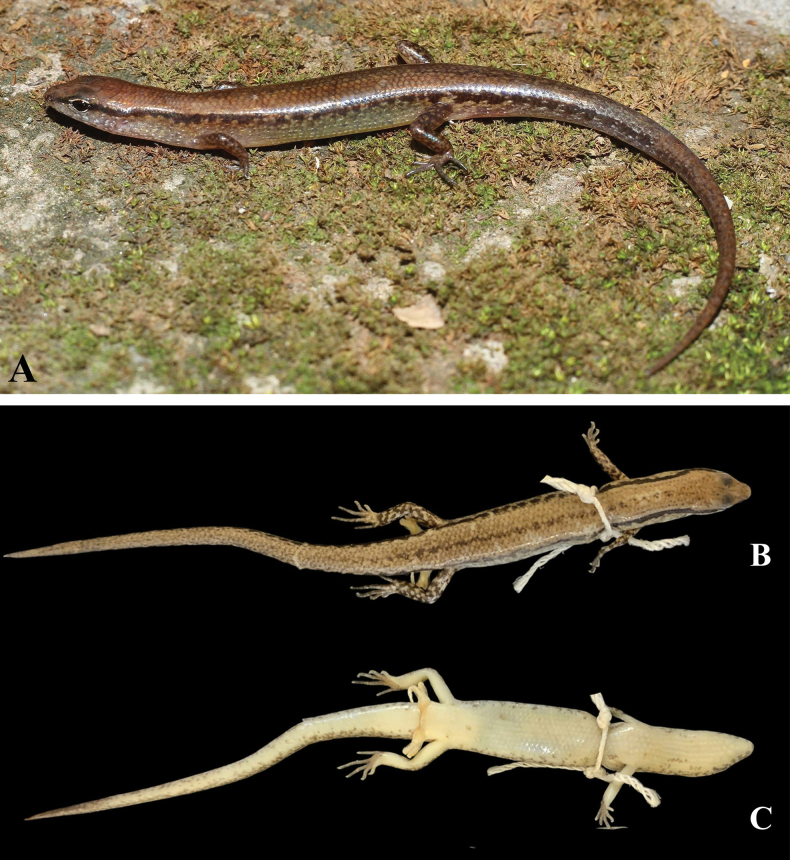
Holotype of *Sphenomorphus tamchucensis* sp. nov. (IB R. 6455), adult male. **A**. In life; **B**. In preservative, dorsal view; **C**. In preservative, ventral view.

**Figure 4.**
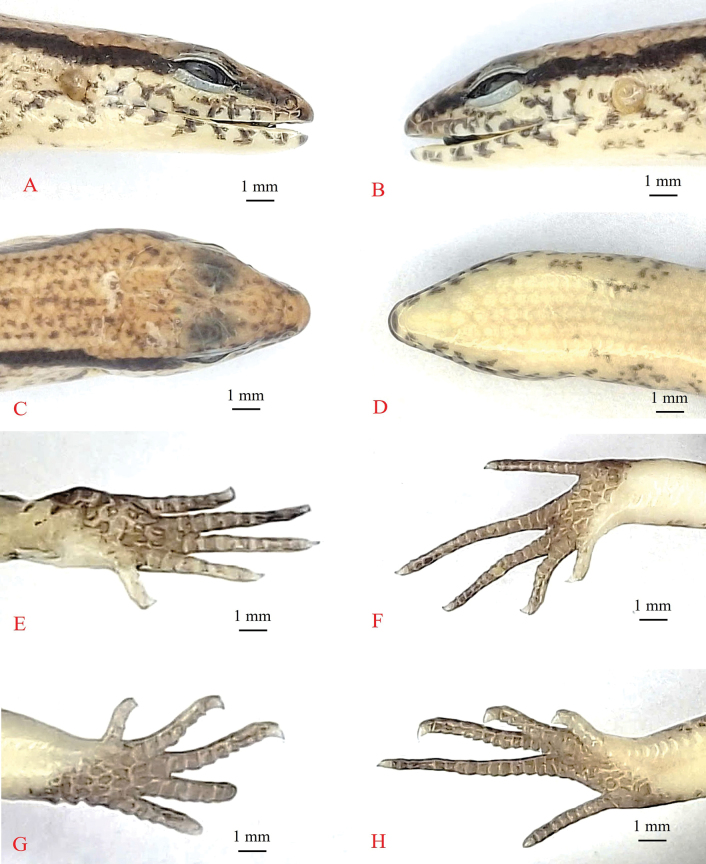
Holotype of *Sphenomorphus tamchucensis* sp. nov. (IB R. 6455). **A–D**. Head; **A, B**. Lateral view; **C**. Dorsal view; **D**. Ventral view; **E, G**. Hands; **F, H**. Feet. Photographs: A.V. Pham.

***Holotype***. IB R.6455 (field number TC-HN. 2025.109) (Figs 3A–C, 4A–H), adult male, collected on 24 April 2025 by T. Q. Nguyen, C.T. Pham, A.V. Pham, and T.Q. Phan in karst forest (20°29'55.4"N, 105°48'29.6"E, at an elevation of 210 m a.s.l.), in the Tam Chuc forest, within Kim Bang Proposed Species and Habitat Conservation Area, Ninh Binh Province, Vietnam.

***Paratypes***. IB R.6456 (field number TC-HN 2025.46), adult male; IB R.6457 (field number TC-HN 2025.108), adult female; IB R.6458 (field number TC-HN 2025.110), adult male; IB R.6459 (field number TC-HN 2025.112), adult male; IB R.6460 (field number TC-HN 2025.121), adult male; IB R.6461 (field number TC-HN 2025.141), adult male; IB R.6462 (field number TC-HN 2025.142), adult female; IB R.6463 (field number TC-HN 2025.143), adult female; IB R.6464 (field number TC-HN 2025.145), adult female; IB R.6465 (field number TC-HN 2025.147), adult female, bear the same data as the holotype.

##### Diagnosis.

The new species can be distinguished from other species of *Sphenomorphus* by a combination of the following characteristics: size small (SVL up to 41.6 mm); primary temporals two; external ear opening without lobules; loreals two; supralabials seven; infralabials six; nuchals absent; midbody scales in 28 rows; dorsal scales smooth, in six rows across the back; paravertebral scales 58–63, not widened; ventral scales in 55–61 rows; 8–10 smooth lamellae beneath finger IV and 13–15 beneath toe IV; toes not reaching to fingers when limbs adpressed along body; dorsal surface of body and tail bronze brown with many tiny dark dots and a discontinuous dark vertebral stripe, from middle of neck to tail base; a black stripe, in two scales wide, running from nostril to eye and extending from posterior margin of eye along upper part of flank and middle of tail.

##### Description of holotype.

Size small (SVL 40.1 mm), tail complete (TaL 51.5 mm, TaL/SVL 1.28); head longer than wide (HL 7.2 mm, HW 5.0 mm, HL/SVL 0.18, HW/SVL 0.12); AG/SVL 0.59, FIL/SVL 0.22, HIL/SVL 0.32; for additional measurements see Table 1. Snout obtuse, anteriorly round; rostral wider than high, distinctly visible from above; supranasals absent; frontonasal wider than long, in contact with rostral, nasals, anterior loreals, and prefrontals; prefrontals in medially contact with each other; frontal posteriorly narrowing, as long as distance to the snout, in contact with prefrontals, first and second supraoculars, and frontoparietals; frontoparietals in contact with each other, and bordered by frontal, three posterior supraoculars, parietals, and interparietal; interparietal lozenge-shaped with a small transparent spot in posterior angle; parietals in contact posteriorly, posterolateral border surrounded by seven scales; nuchal scales absent; nostril in centre of nasal; postnasals absent; loreals two, anterior higher but narrower than posterior, posterior loreal in contact with anterior loreal, prefrontal, anterior supraciliary, preoculars, and second supralabials; preoculars two; presuboculars two, posterior scale in contact with second and third supralabials; supraciliaries eight, first largest, first to third in contact with first supraocular, fourth and fifth in contact with second supraocular, sixth and seventh in contact with third supraocular, eighth in contact with fourth supraocular; supraoculars four, third widest, fourth supraocular followed by a small scale; postocular single; postsuboculars five, lower one in contact with sixth supralabial; primary temporals two, lower one in contact with sixth and seventh supralabials; secondary temporals two, upper one very large, overlapped by lower one, in contact with parietal; lower eyelid moveable, scaly, separated from supralabials by one row of small scales; supralabials seven, sixth enlarged, fifth below the eye; external ear opening nearly round, smaller than eye length, without lobules; tympanum sunk; infralabials six, first very small; mental wider than long, round anteriorly, in contact with anterior infralabial on each side and postmental; postmental undivided, in contact with mental, first and second infralabials, and anterior pair of chinshields; three pairs of chinshields, anterior pair in contact with each other anteriorly, second pair separated from each other by a gular scale, and posterior pair separated from each other by three scales; midbody scales in 28 rows; dorsal scales smooth, subequal to lateral and ventral scales, 1/2 + 6 + 1/2 scale rows between dark stripes on upper lateral zones; paravertebral scales 62, not widened; ventrals smooth, in 58 rows; precloacals four, inner scales overlapping outer ones, medial two enlarged, right scale overlapped by left scale; everted hemipenis bifurcated; tai thick at base, median subcaudals on posterior part of tail slightly widened. Limbs well developed, pentadactyl; third and fourth fingers equal in length; subdigital lamellae smooth, numbering 8/9 under fourth finger and 15/15 under fourth toe; toe and finger separated when adpressed along body, adpressed forelimb reaching to eye (Table 1).

**Table 1.** Morphological characteristics of the type series of *Sphenomorphus tamchucensis* sp. nov. from Ninh Binh Province, Vietnam.

	IB R.6455	IB R.6458	IB R.6460	IB R.6459	IB R.6461	IB R.6456	IB R.6462	IB R.6457	IB R.6463	IB R.6464	IB R.6465	Min-Max
Type status	Holotype	Paratype	Paratype	Paratype	Paratype	Paratype	Paratype	Paratype	Paratype	Paratype	Paratype	
Sex	Male	Male	Male	Male	Male	Male	Female	Female	Female	Female	Female	
**Measurements** (in mm)												
SVL	40.1	41.6	41.5	37.0	41.0	37.3	37.6	39.9	39.8	36.3	39.0	36.3–41.6
TaL (*generated)	51.5*	37.8*	47.1*	55.8	52.8	43.9*	33.4*	39.5*	50.1	25.6*	33.8*	50.1–55.8
AG	23.6	23.3	23.8	19.8	23.9	20.0	20.1	22.0	21.1	19.0	20.0	19.0–23.8
SL	3.0	3.0	3.1	2.9	3.1	2.8	2.8	3.1	2.7	2.6	2.9	2.6–3.1
STL	7.5	8.1	8.3	7.3	7.6	7.3	7.2	7.5	7.4	7.0	7.8	7.0–8.3
SFlL	13.6	13.9	14.8	12.7	14.0	12.6	12.4	13.5	13.5	13.0	12.6	12.4–14
END	1.2	1.3	1.3	1.2	1.2	1.1	1.0	1.0	1.1	1.0	1.0	1.0–1.3
EL	2.0	2.2	2.2	2.0	2.2	2.0	2.0	2.1	2.1	2.0	2.0	2.0–2.2
HL	7.2	7.7	7.6	6.9	7.34	7.0	7.0	7.1	7.1	6.9	7.1	6.9–7.6
HW	5.0	4.6	5.4	4.8	5.3	4.5	4.8	5.0	3.7	4.3	4.6	3.7–5.3
HH	4.0	3.9	4.2	3.8	4.1	3.8	3.8	3.9	3.7	3.4	3.4	3.4–4.2
TYD	1.1	1.1	1.1	1.1	1.1	1.0	1.1	1.1	1.1	1.0	1.1	1.0–1.1
FlL	8.9	8.7	9.2	8.2	9.2	8.1	8.14	8.2	9.1	8.4	8.1	8.1–9.1
HlL	13.0	13.9	14.0	11.9	13.5	12.0	11.9	13.0	13.3	12.7	12.8	11.9–14.0
FIL/SVL	0.22	0.21	0.22	0.22	0.22	0.22	0.22	0.21	0.23	0.23	0.21	0.21–0.23
HIL/SVL	0.32	0.33	0.34	0.32	0.33	0.32	0.32	0.33	0.33	0.34	0.33	0.32–0.34
NS	7	7	6	8	8	8	5	7	7	8	8	7–8
So	4	4	4	4	4	4	4	4	4	4	4	4–4
Nu	0	0	0	0	0	0	0	0	0	0	0	0
Lo	2	2	2	2	2	2	2	2	2	2	2	2–2
PC	2	2	2	2	2	2	2	2	2	2	2	2–2
PS	2	2/1	2	2	2	1	2	1	1	2	2	1–2
SC	7/7	7/7	7/8	8/7	8/8	7/7	7/7	7/7	8/8	7/7	7/7	7–8
Po	1	1	1	1	1	1	1	1	1	1	1	1–1
Pos	5	5	5	5	5	3	5	5	3	3	3	3–5
PT	2	2	2	2	2	2	2	2	2	2	2	2–2
ST	2	2	2	2	2	2	2	2	2	2	2	2–2
SL	7/7	7/7	7/7	7/7	7/7	7/7	7/7	7/7	7/7	7/7	7/7	7–7
AL	0	0	0	0	0	0	0	0	0	0	0	0–0
IF	6/6	6/6	6/6	6/6	6/6	6/6	6/6	6/6	6/6	6/6	6/6	6–6
CS	3	3	3	3	3	3	3	3	3	3	3	3–3
MB	28	28	28	28	28	28	28	28	28	28	28	28–28
PV	62	62	62	58	63	63	62	62	60	63	62	58–63
VR	58	56	56	56	56	60	58	56	58	61	59	56–61
PR	2	2	2	2	2	2	2	2	2	2	2	2–2
FL4	8/9	10/10	10/10	9/10	10/9	9/10	10/10	8/9	9/10	10/10	10/10	8–10
TL4	15/15	13/13	15/14	15/15	14/13	15/15	15/14	13/8+	15/15	15/14	15/15	13–15
MB	28	28	28	28	28	28	28	28	28	28	28	28–28
PV	62	62	62	58	63	63	62	62	60	63	62	58–63

##### Colouration in preservation.

Dorsal surface of body and tail bronze brown with many tiny dark dots and a discontinuous dark vertebral stripe, from middle of neck to tail base; a black stripe, in two scales wide, running from nostril to eye and extending from posterior margin of eye along upper part of flank and middle of tail, paler in posterior part of tail; supralabials and infralabials with dark bars on sutures; lateral side of the head and flank light brown, with a few dark spots; upper of limbs brown with light spots; chin, throat, venter, tail base, underside of fore and hind limbs cream; underside of tail tip with very small, grey-brown dots (Figs 3B, 3C, 4A–H). For colouration in life, see Fig. 3A.

##### Variation.

Paravertebral scales 58 in IB R.6459; 63 in IB R.6456, IB R.6461, IB R.6464; 60 in IB R.6463; ventrals in 56 transverse rows in IB R.6457, IB R.6458, IB R.6459, IB R.6460; 60 in IB R.6456, 59 in IB R.6465. For measurements and scale counts of the type series, see Table 1.

##### Distribution.

*Sphenomorphus tamchucensis* sp. nov. is currently known only from the type locality in Kim Bang SHCA, Ninh Binh Province, Vietnam (Suppl. material 1).

##### Natural history.

Specimens were found on the ground in secondary forest between 18:30 and 22:00. The surrounding habitat was evergreen forest with medium-sized and small hardwoods mixed with shrubs. Air temperatures at the site ranged from 25–33 °C and relative humidity was 60–75%. Other reptile species encountered included *Acanthosaura lepidogaster* (Cuvier, 1829).

##### Etymology.

The specific epithet *tamchucensis* refers to the type locality, Tam Chuc forest within Kim Bang SHCA, Ninh Binh Province, where the new species was discovered. We recommend “Tam Chuc’s Smooth Skink” as the common English name and “Thằn lằn phê nô tam chúc” as the Vietnamese name.

##### Comparisons.

We compare the new species with other known taxa in the genus *Sphenomorphus* in the mainland Indochinese region: Vietnam, Laos, Myanmar, southern China, Cambodia, Thailand, and Peninsular Malaysia. Morphological comparisons were based on data from Duméril and Bibron (1839), Boulenger (1890), van Denburgh (1912), Smith (1924), Smith (1935), Taylor (1963), Eremchenko (2003), Grismer and Quah (2015), Nguyen et al. (2013), Sumarli et al. (2016), Nguyen et al. (2018), Grismer et al. (2019), Grismer et al. (2020), Le et al. (2020), and Bragin et al. (2025).

Morphologically, *Sphenomorphus tamchucensis* sp. nov. resembles *S. tonkinensis*. However, the new species can be distinguished from *S. tonkinensis* by having a smaller FIL/SVL ratio (0.21–0.23 vs 0.24–0.26), HIL/SVL (0.32–0.34 vs 0.35–0.39), fewer midbody scale rows (28 vs 32–34), fewer supraciliaries (7–8 vs 9), fewer paravertebral scales (58–63 vs 65–72), fewer lamellae under toe IV (13–15 vs 15–19), fewer lamellae under finger IV (8–10 vs 10–11), and fewer dorsal scale rows on back (6 vs 8).

*Sphenomorphus tamchucensis* sp. nov. differs from *S. annamiticus* in having more midbody scale rows (28 vs 24), fewer lamellae under toe IV (13–15 vs 17–19), fewer lamellae under finger IV (8–10 vs 11–14); from *S. anomalopus* (Boulenger, 1890) in having fewer midbody scale rows (28 vs 38) and a smaller size (SVL 36.3–41.5 mm vs 70 mm); from *S. bacboensis* in having fewer midbody scale rows (28 vs 30–32), and more supralabials (7 vs 6); from *S. buenloicus* in having fewer midbody scale rows (28 vs 32–34), fewer lamellae under toe IV (13–15 vs 16–19), more anterior temporals (2 vs 1), and a smaller size (SVL 36.3–41.5 mm vs 56 mm); from *S. cameronicus* Smith, 1924 in having a smaller size (SVL 36.3–41.5 mm vs 70 mm) and fewer midbody scale rows (28 vs 38); from *S. cryptotis* in having a smaller size (SVL 36.3–41.5 mm vs 58–79 mm), fewer midbody scale rows (28 vs 32–38), fewer lamellae under toe IV (13–15 vs 17–23), and fewer paravertebral scale rows (58–63 vs 71–89); from *S. grandisonae* Taylor, 1962 in having a larger size (SVL 36.3–41.5 mm vs 30 mm) and more lamellae under toe IV (13–15 vs 12); from *S. helenae* Cochran, 1927 in having fewer midbody scale rows (28 vs 30) and the presence of an interrupted (vs continuous) lateral stripe; from *S. incognitus* in having a smaller size (SVL 36.3–41.5 mm vs 80–103 mm), fewer midbody scale rows (28 vs 36–40), fewer paravertebral scale rows (58–63 vs 67–80), and fewer lamellae under toe IV (13–15 vs 19–24); from *S. indicus* in having a smaller size (SVL 36.3–41.5 mm vs 61–90 mm), fewer paravertebral scale rows (58–63 vs 65–77), and fewer lamellae under toe IV (13–15 vs 16–20); from *S. lineopunctulatus* in having a smaller size (SVL 36.3–41.5 mm vs 84 mm), fewer midbody scale rows (28 vs 38), and fewer paravertebral scale rows (61–69 vs 76); from *S. maculatus* (Blyth, 1853) in having a smaller size (SVL 36.3–41.5 mm vs 62 mm), fewer midbody scale rows (28 vs 38–42), fewer paravertebral scale rows (58–63 vs 69–78), and fewer lamellae under toe IV (13–15 vs 18–21); from *S. malayanus* (Doria, 1888) in having fewer ventral scales (55–61 vs 74), and fewer paravertebral scales (58–63 vs 76–80), and deeply sunk (vs shallow) tympanum; from *S. mimicus* Taylor, 1962 in having fewer midbody scale rows (28 vs 30), fewer supraciliaries (7–8 vs 9), and few lamellae under fourth toe (13–15 vs 16); from *S. orientale* (Shreve, 1940) in having more midbody scale rows (28 vs 24–26) and fewer paravertebral scale rows (58–63 vs 69–71); from *S. praesignis* (Boulenger, 1900) in having a smaller size (SVL 36.3–41.5 mm vs 109 mm) and fewer lamellae under finger IV (8–10 vs 12–15); from *S. phuquocensis* in having a smaller size (SVL 36.3–41.5 mm vs 60 mm), more midbody scale rows (28 vs 23), fewer lamellae under finger IV (8–10 vs 14), and fewer lamellae under toe IV (13–15 vs 18 or 19); from *S. preylangensis* Grismer et al., 2019 in having a smaller size (SVL 36.3–41.5 mm vs 51.4–87.6 mm), more midbody scale rows (28 vs 24), fewer lamellae under finger IV (8–10 vs 11–14), and fewer lamellae under toe IV (13–15 vs 17–19); from *S. sanctus* (Duméril & Bibron, 1839) in having a fewer paravertebral scales (58–63 vs 71), fewer supraoculars (4 vs 5), and fewer lamellae under toe IV (13–15 vs 26–27); from *S. scotophilus* (Boulenger, 1900) in having fewer supraoculars (4 vs 5) and fewer lamellae under fourth toe (13–15 vs 22–23); from *S. senja* Grismer & Quah, 2015 in having a smaller size (SVL 36.3–41.5 mm vs 60–65 mm), fewer paravertebral scales (58–63 vs 72–73), and fewer ventral scale rows (60–67 vs 68); from *S. shelfordi* (Boulenger, 1900) in having a smaller size (SVL 36.3–41.5 mm vs 67 mm), fewer lamellae under toe IV (13–15 vs 28–29), and the absence of nuchals (vs presence of a single pair of nuchals); from *S. stellatus* in having a smaller size (SVL 36.3–41.5 mm vs 80 mm), more midbody scale rows (28 vs 24) and the absence (vs present) of two enlarged, broader than long, vertebral scale rows; from *S. sungaicolus* Sumarli et al., 2016 in having a smaller size (SVL 36.3–41.5 mm vs 67–90 mm), fewer midbody scale rows (28 vs 39–44), fewer paravertebral scales (58–63 vs 72–81), and fewer ventral scale rows (56–61 vs 74–86); from *S. tersus* (Smith, 1916) in having a smaller size (SVL 36.3–41.5 mm vs 90–92 mm) and two loreals (vs three); from *S. tetradactylus* in having more midbody scale rows (28 vs 20), forelimb with five digists (vs with four digits), and the absence (vs present) of external ear opening; from *S. tridigitus* in having more midbody scale rows (28 vs 18-20), more paravertebral scales (58–63 vs 52), more ventral scales (55 -61 vs 52), forelimb with five digits (vs with three digits),and more lamellae under fourth toe (13–15 vs 7-8); from *S. tritaeniatus* in having a smaller size (SVL 36.3–41.5 vs 48.8 mm), fewer midbody scale rows (28 vs 38), and fewer paravertebral scales (58–63 vs 81); from *S. valentinae* in having fewer anterior temporals (2 vs 3), more infralabials (6 vs 4), more midbody scale rows (28 vs 18), more lamellae under finger IV (8–10 vs 5), and more lamellae under toe IV (13–15 vs 6); from *S. veunsaiense* in having a larger size (SVL 36.3–41.5 mm vs 33.6–35.2 mm), more midbody scale rows (28 vs 20–22), more paravertebral scales (58–63 vs 51–53), and more lamellae under fourth toe (13–15 vs 6); from *S. yersini* in having a smaller size (SVL 36.3–41.5 mm vs 50–56 mm), more paravertebral scales (58–63 vs 50–55), more anterior temporals (2 vs 1), fewer midbody scale rows (28 vs 32–34), and fewer lamellae under toe IV (13–15 vs 18–20).

## Discussion

Over the last 10 years, seven additional species have been described within the genus *Sphenomorphus* (Uetz et al. 2025). Three of the species—*S. phuquocensis*, *S. valentinae*, and *S. yersini*—were newly discovered in Vietnam (Nguyen et al. 2018; Grismer et al. 2020; Bragin et al. 2025). Our discovery brings the species number of *Sphenomorphus* in Vietnam to 18. In terms of morphology, *S. tamchucensis* sp. nov. closely resembles *S. tonkinensis*, while in the phylogenetic analysis, the new species was recovered as an independent lineage with no clear sister taxon.

Although northern Vietnam harbours one of the most extensive limestone karst systems in the world (Clements et al. 2006), its biodiversity remains poorly understood. Pham et al. (2024) recently discovered a new species of *Scincella* from the limestone karst forest of Phu Tho Province. Our new finding further underscores the ecological significance of these karst landscapes in sheltering and sustaining unique biodiversity and additional field studies in this region is likely to the discovery of new reptile taxa.

The new species is currently known only from the proposed Kim Bang Species and Habitat Conservation Area, Ninh Binh Province. It has a small distribution range with an estimated area of less than 30 km^2^, which has been experiencing severe habitat degradation because of road construction and limestone quarrying. We recommend listing the species as Data Deficient based on the IUCN Red List categories and criteria (IUCN 2025). Further research is needed to clarify the population status of this species and to determine specific anthropogenic threats at the site.

## Supplementary Material

XML Treatment for Sphenomorphus tamchucensis

## Additional information

### Use of AI

No use of AI was reported.

### Author ORCIDs

Anh Van Pham https://orcid.org/0000-0002-6023-3418

Cuong The Pham https://orcid.org/0000-0001-5158-4526

Linh Thuy Ha https://orcid.org/0009-0006-3290-9261

Minh Duc Le https://orcid.org/0009-0000-5097-8531

Tien Quang Phan https://orcid.org/0000-0002-2738-5364

Anh Ngoc Thi Ho https://orcid.org/0009-0009-0711-811X

Minh Le https://orcid.org/0000-0002-2953-2815

Truong Quang Nguyen https://orcid.org/0000-0002-6601-0880

### Data availability

All of the data that support the findings of this study are available in the main text or Supplementary Information.

## Supplementary materials

Supplementary material 1 The type locality of *Sphenomorphus tamchucensis* sp. nov. https://doi.org/10.3897/zookeys.1266.176724.suppl1 Data type doc https://binary.pensoft.net/file/1510771This dataset is made available under the Open Database License (http://opendatacommons.org/licenses/odbl/1.0/). The Open Database License (ODbL) is a license agreement intended to allow users to freely share, modify, and use this Dataset while maintaining this same freedom for others, provided that the original source and author(s) are credited. Anh Van Pham, Cuong The Pham, Linh Thuy Ha, Minh Duc Le, Tien Quang Phan, Anh Ngoc Thi Ho, Minh Le, Truong Quang Nguyen

Supplementary material 2 Phylogenetic, genomic & statistics https://doi.org/10.3897/zookeys.1266.176724.suppl2 Data type docx https://binary.pensoft.net/file/1510772This dataset is made available under the Open Database License (http://opendatacommons.org/licenses/odbl/1.0/). The Open Database License (ODbL) is a license agreement intended to allow users to freely share, modify, and use this Dataset while maintaining this same freedom for others, provided that the original source and author(s) are credited. Anh Van Pham, Cuong The Pham, Linh Thuy Ha, Minh Duc Le, Tien Quang Phan, Anh Ngoc Thi Ho, Minh Le, Truong Quang Nguyen

## References

[B1] Boulenger GA (1890) First report on additions to the lizard collection in the British Museum (Natural History). Proceedings of the Zoological Society of London 1890: 77–86.

[B2] Bragin AM, Geissler P, Trofimets AV, Neang T, Le XS, Nguyen VT, Poyarkov NA (2025) A new species of skink from mountains of southern Vietnam (Reptilia, Squamata, Scincidae). Current Studies in Herpetology 25(1/2): 3–21. 10.18500/1814-6090-2025-25-1-2-3-21

[B3] Chan KO, Grismer LL (2025) GroupStruct2: A user-friendly graphical user interface for statistical and visual support in species diagnosis. Systematic Biology: syaf090. 10.1093/sysbio/syaf090 PMC13291812 41427886

[B4] Clements R, Sodhi NS, Schilthuizen M, Ng PKL (2006) Limestone karsts of Southeast Asia: Imperiled arks of biodiversity. Bioscience 56(9): 733–742. 10.1641/0006-3568(2006)56[733:LKOSAI]2.0.CO;2

[B5] Darriba D, Taboada GL, Doallo R, Posada D (2012) jModelTest 2: More models, new heuristics and parallel computing. Nature Methods 9(8): 772–772. 10.1038/nmeth.2109 PMC4594756 22847109

[B6] Duméril AMC, Bibron G (1839) Erpétologie Générale ou Histoire Naturelle Complète des Reptiles, Vol. 5. Roret/Fain et Thunot, Paris, 871 pp.

[B7] Eremchenko VK (2003) Generic and specific redefinition and redescription of the North-Vietnam skink (*Scincella melanosticta* (Boulenger, 1887)). Izvestiya Vuzov (= Proceedings of Universities and Institutes) 2: 20–28.

[B8] Folmer O, Black M, Hoeh W, Lutz R, Vrijenhoek R (1994) DNA primers for amplification of mitochondrial cytochrome c oxidase subunit I from diverse metazoan invertebrates. Molecular Marine Biology and Biotechnology 3: 294–299. 10.1371/journal.pone.0013102 7881515

[B9] Grismer LL, Quah ESH (2015) The Rediscovery of *Sphenomorphus* Doria, 1888 (Squamata: Scincidae) from the Titiwangsa Mountain Range of Peninsular Malaysia and its re-description as *S. senja* sp. nov. Zootaxa 3931(1): 63–70. 10.11646/zootaxa.3931.1.4 25781814

[B10] Grismer LL, Perry LJW, Quah ESH, Anuar S, Poyarkov NA, Thy N, Orlov NL, Thammachoti P, Seiha H (2019) Integrative taxonomy of the Asian skinks *Sphenomorphus stellatus* (Boulenger, 1900) and *S. praesignis* (Boulenger, 1900) with the resurrection of *S. annamiticus* (Boettger, 1901) and the description of a new species from Cambodia. Zootaxa 4683(3): 381–411. 10.11646/zootaxa.4683.3.4 31715918

[B11] Grismer LL, Nazarov RA, Bobrov VV, Poyarkov NA (2020) A new species of *Sphenomorphus* (Squamata: Scincidae) from Phu Quoc Island, Vietnam with a discussion of biogeography and character state evolution in the *S. stellatus* group. Zootaxa 4801(3): 461–487. 10.11646/zootaxa.4801.3.3 33056644

[B12] Hillis DM, Bull JJ (1993) An empirical test of bootstrapping as a method for assessing confidence in phylogenetic analysis. Systematic Biology 42(2): 182–192. 10.1093/sysbio/42.2.182

[B13] IUCN (2025) The IUCN Red List of Threatened Species. Version 2025-2. https://www.iucnredlist.org

[B14] Jeong TJ, Jun J, Han S, Kim HT, Oh K, Kwak M (2013) DNA barcode reference data for the Korean herpetofauna and their applications. Molecular Ecology Resources 13(6): 1019–1032. 10.1111/1755-0998.12055 23311467

[B15] Jombart T, Devillard S, Balloux F (2010) Discriminant analysis of principal components: a new method for the analysis of genetically structured populations. BMC genetics 11: 94. 10.1186/1471-2156-11-94 PMC2973851 20950446

[B16] Kearse M, Moir R, Wilson A, Stones-Havas S, Cheung M, Sturrock S, Buxton S, Cooper A, Markowitz S, Duran C, Thierer T, Ashton B, Mentjes P, Drummond A (2012) Geneious Basic: An integrated and extendable desktop software platform for the organization and analysis of sequence data. Bioinformatics (Oxford, England) 28(12): 1647–1649. 10.1093/bioinformatics/bts199 PMC3371832 22543367

[B17] Le MV, Nguyen LT, Vo BD, Murphy RW, Nguyen VDH, Nguyen SN (2020) A review of the genus *Sphenomorphus* Fitzinger, 1843 (Squamata: Scincidae) in southern Vietnam, with additional data on *S. sheai* and *S. tridigitus*. VNUHCM Journal of Science and Technology Development 23(1): 470–478. 10.32508/stdj.v23i1.1733

[B18] Lleonart J, Salat J, Torres GJ (2000) Removing allometric effects of body size in morphological analysis. Journal of Theoretical Biology 205: 85–93. 10.1006/jtbi.2000.2043 10860702

[B19] Nguyen TQ (2011) Systematics, ecology, and conservation of the lizard fauna in northeastern Vietnam, with special focus on the genera *Pseudocalotes* (Agamidae), *Goniurosaurus* (Eublepharidae), *Sphenomorphus* and *Tropidophorus* (Scincidae) from this country. PhD Thesis. University of Bonn.

[B20] Nguyen TQ, Schmitz A, Nguyen TT, Orlov NL, Böhme W, Ziegler T (2011) Review of the genus *Sphenomorphus* Fitzinger, 1843 (Squamata: Sauria: Scincidae) in Vietnam, with description of a new species from northern Vietnam and southern China and the first record of *Sphenomorphus mimicus* Taylor, 1962 from Vietnam. Journal of Herpetology 45(2): 145–154. 10.1670/09-068.1

[B21] Nguyen TQ, Nguyen KV, Devender RWV, Bonkowski M, Ziegler T (2013) A new species of *Sphenomorphus* Fitzinger, 1843 (Squamata: Sauria: Scincidae) from Vietnam. Zootaxa 3734(1): 56–62. 10.11646/zootaxa.3734.1.6 25277895

[B22] Nguyen LT, Schmidt HA, Haeseler A, Bui MQ (2015) IQ-TREE: A fast and effective stochastic algorithm for estimating maximum-likelihood phylogenies. Molecular Biology and Evolution 32(1): 268–274. 10.1093/molbev/msu300 PMC4271533 25371430

[B23] Nguyen SN, Nguyen LT, Nguyen VDH, Orlov NL, Murphy RW (2018) A new skink of the genus *Sphenomorphus* Fitzinger, 1843 (Squamata: Scincidae) from Hon Ba Nature Reserve, southern Vietnam. Zootaxa 4438(2): 313–326. 10.11646/zootaxa.4438.2.6 30313147

[B24] Pham AV, Pham CT, Le MD, Ngoc HN, Ziegler T, Nguyen TQ (2024) A new skink of the genus *Scincella* Mittleman, 1950 (Squamata: Scincidae) from Hoa Binh Province, northern Vietnam. Zootaxa 5428(1): 91–106. 10.11646/zootaxa.5428.1.4

[B25] R Core Team (2025) R: A language and environment for statistical computing. R Foundation for Statistical Computing. Vienna. 2018. http://www.R–project.org [Accessed on 24 December 2025]

[B26] Ronquist F, Teslenko M, Mark P, Ayres DL, Darling A, Höhna S, Larget B, Liu L, Suchard MA, Huelsenbeck JP (2012) MrBayes 3.2: efficient Bayesian phylogenetic inference and model choice across a large model space. Systematic Biology 61: 539 542. 10.1093/sysbio/sys029 PMC3329765 22357727

[B27] Simmons JE (2002) Herpetological collecting and collections management. Herpetological Circular 31: 1–153.

[B28] Smith MA (1924) Two new lizards and a new tree frog from the Malay Peninsula. Journal of the Federated Malay States Museum 11: 183–186.

[B29] Smith MA (1935) The Fauna of British India, Including Ceylon and Burma. Reptilia and Amphibia II. Taylor And Francis, London, 440 pp.

[B30] Sumarli A, Grismer LL, Wood Jr PL, Ahmad AB, Rizal S, Ismail LH, Izam NAM, Ahmad N, Linkem CW (2016) The first riparian skink (Genus: *Sphenomorphus* Strauch, 1887) from Peninsular Malaysia and its relationship to other Indochinese and Sundaic species. Zootaxa 4173(1): 29–44. 10.11646/zootaxa.4173.1.3 27701201

[B31] Swofford DL (2001) PAUP*. Phylogenetic Analysis Using Parsimony (* and Other Methods), Version 4. Sinauer Associates, Sunderland, Massachusetts.

[B32] Taylor EH (1963) The lizards of Thailand. The University of Kansas Science Bulletin 44: 687–1077.

[B33] Thompson JD, Higgins DG, Gibson TJ (1997) CLUSTAL W: Improving the sensitivity of progressive multiple sequence alignment through sequence weighting, position-specific gap penalties and weight matrix choice. Nucleic Acids Research 22(22): 4673–4680. 10.1093/nar/22.22.4673 PMC308517 7984417

[B34] Thorpe RS (1975) Quantitative handling of characters useful in snake systematics with particular reference to interspecific variation in the Ringed Snake *Natrix natrix* (L.). Biological Journal of the Linnean Society 7: 27–43. 10.1111/j.1095-8312.1975.tb00732.x

[B35] Thorpe RS (1983) A review of the numerical methods for recognizing and analyzing racial differentiation. In: Felsenstein J (Ed.) Numerical Taxonomy. NATO ASI Series G: Ecological Sciences. Springer-Verlag, Berlin, 404–423. 10.1007/978-3-642-69024-2_43

[B36] Turan C (1999) A note on the examination of morphometric differentiation among fish populations: The Truss System. Turkish Journal of Zoology 23: 259–263.

[B37] Uetz P, Freed P, Hošek J [Eds] (2025) The Reptile Database. http://www.reptile-database.org/ [Accessed on 23 December 2025]

[B38] Van Denburgh J (1912) Concerning certain species of reptiles and amphibians from China, Japan, the Loo Choo Islands, and Formosa. Proceedings of the California Academy of Science 4: 187–258.

