# Supplementary material for: Sphenomorphus tamchucensis sp. nov. (Squamata, Scincidae), a new skink from Vietnam
Source: Zookeys. 2026 Jan 13;1266:263–79. doi: 10.3897/zookeys.1266.176724 (PMC12820565; doi:10.3897/zookeys.1266.176724)
Supplement: Supplementary material 1 — The type locality of Sphenomorphus tamchucensis sp. nov. [file zookeys-1266-263_article-176724__-s001.doc]

***Sphenomorphus tamchucensis* sp. nov. (Squamata, Scincidae), a new skink from Vietnam**


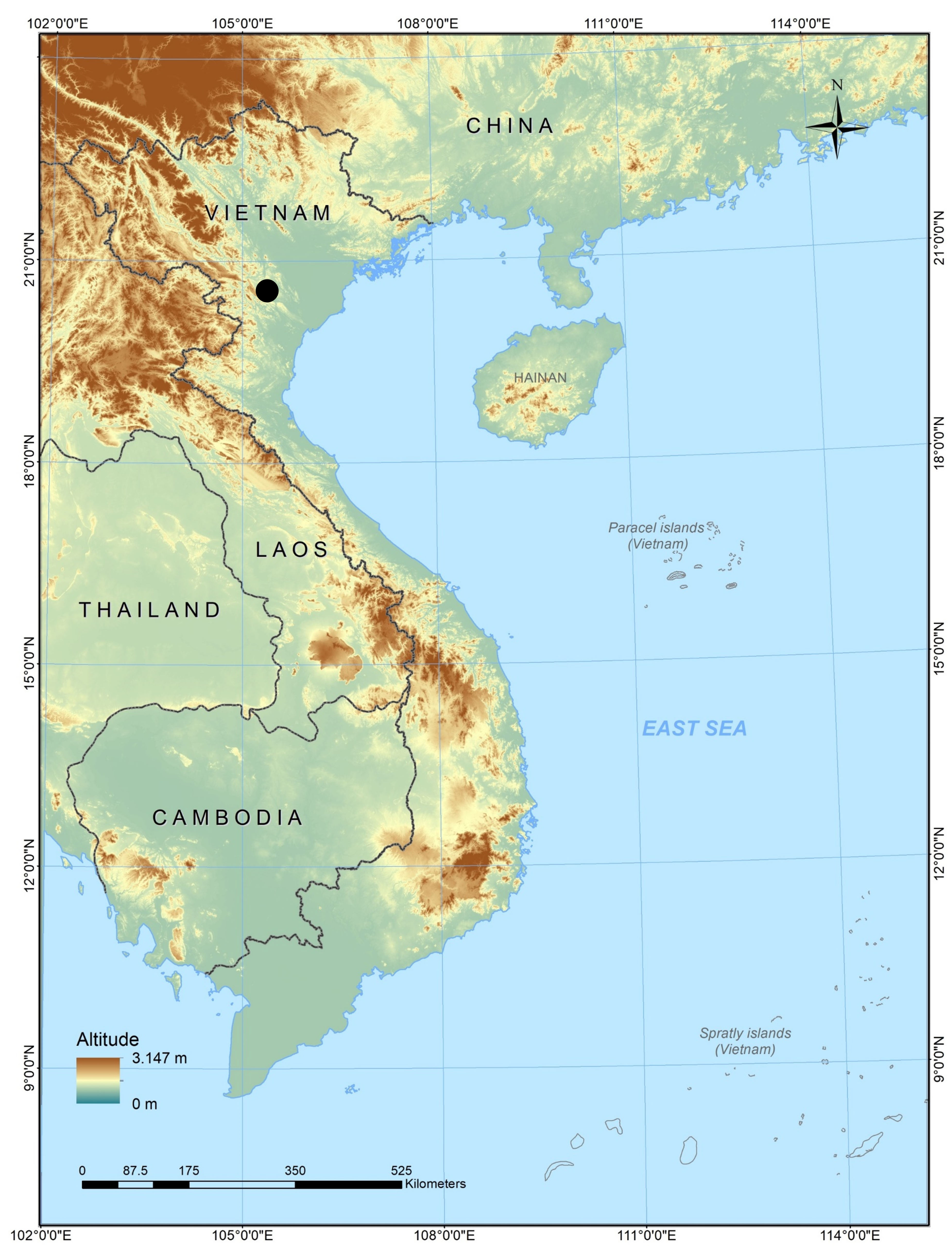


**Figure S1.** The type locality of *Sphenomorphus tamchucensis* sp. nov. in Ninh Binh Province, Vietnam.
