# Supplementary material for: Sphenomorphus tamchucensis sp. nov. (Squamata, Scincidae), a new skink from Vietnam
Source: Zookeys. 2026 Jan 13;1266:263–79. doi: 10.3897/zookeys.1266.176724 (PMC12820565; doi:10.3897/zookeys.1266.176724)
Supplement: Supplementary material 2 — Phylogenetic, genomic & statistics [file zookeys-1266-263_article-176724__-s002.docx]

***Sphenomorphus tamchucensis* (Squamata, Scincidae), a new skink from Vietnam**

**Tables**

**Table S1**. Samples used in this study

| **Voucher/Field number** | **Species** | **Locality** | **GenBank accession no.** | **References** |  |
| --- | --- | --- | --- | --- | --- |
|  |  |  |  |  |  |
| **IB R. 6455** | ***S. tamchucensis* sp. nov.** | **Ninh Binh, Vietnam** |  | **This study** |  |
| **IB R. 6458** | ***S. tamchucensis* sp. nov.** | **Ninh Binh, Vietnam** |  | **This study** |  |
| USNM594402 | *S. indicus* | Tanintharyi, Ywahilu, Ywahilu, Myanmar | MT608593 | Zug (2022) |  |
| HS17088 | *S. indicus* | Tunxi, Huangshan, Anhui, China | MK450438 | Tang et al. (2019) |  |
| ZMMU R-13680-00094 | *S. maculatus* | Dong Nai Province, Vietnam | MH119629 | Neang et al. (2018) |  |
| USNM: Herp: 587039 | *S. maculatus* | Tanintharyi, Myanmar | MG935700 | Mulcahy et al. (2018) |  |
| ITBCZ 4357 | *S. buenloicus* | Khanh Hoa, Vietnam | MH237969 | Nguyen et al. (2018) |  |
| ITBCZ 5060 | *S. buenloicus* | Khanh Hoa, Vietnam | MH237970 | Nguyen et al. (2018) |  |
| USNM: Herp:585412 | *S. scutatus* | Ngermalk Island, Ngermalk Island, Palau | MH274672 | Mulcahy et al. (2022) |  |
| USNM: Herp:585429 | *S. scutatus* | Ngermalk Island, Ngermalk Island, Palau | MH274671 | Mulcahy et al. (2022) |  |
| USNM: Herp:595507 | *S. tonkinensis* | Hai Phong, Vietnam | OM420325 | Miller et al. (Unpublished) |  |
| USNM: Herp:595508 | *S. tonkinensis* | Hai Phong, Vietnam | OM420326 | Miller et al. (Unpublished) |  |
| NC41124 | *S. incognitus* | Huangshan, Anhui, China | NC41124 | Tang et al. (2019) |  |
| MH329292 | *S. incognitus* | Huangshan, Anhui, China | MH329292 | Tang et al. (2019) |  |
| CIB 119027 | *S. cryptotis* | Sichuan, China | OP942215 | Jia et al. (2024) |  |
| ZMMU_NAP-07141 | *S. veunsaiense* | Gia Lai, Vietnam | PP931018 | Bragin et al. (2025) |  |
| ZMMU_NAP-05515 | *S. veunsaiense* | Gia Lai, Vietnam | PP931016 | Bragin et al. (2025) |  |
| FBRC_DNA207 | *S. dussumier* | Ghats, India | MK089440 | Ganesh et al. (2018) |  |
| ITBCZ 5684 | *S. yersini* | Khanh Hoa, Vietnam | MH237971 | Nguyen et al. (2018) |  |
| ITBCZ 5685 | *S. yersini* | Khanh Hoa, Vietnam | MH237972 | Nguyen et al. (2018) |  |
| USNM:Herp:533601 | *S. solomonis* | Temotu, Solomon Islands | MH274678 | Mulcahy et al. (2018) |  |
| USNM:Herp:533597 | *S. solomonis* | Temotu, Solomon Islands | MH274679 | Mulcahy et al. (2018) |  |
| USNM:Herp:595505 | *S.* sp. | Tuyen Quang, Vietnam | OM420415 | Miller et al. (Unpublished) |  |
| USNM:Herp:595504 | *S.* sp. | Tuyen Quang, Vietnam | OM420414 | Miller et al. (Unpublished) |  |
|  | *Plestiodon elegans* | China | KJ643142 | Chen et al. (2019), (Unpublished) |  |

**Table S2.** Uncorrected (“p”) distance matrix showing percentage pairwise genetic divergence (COI) between the new species (highlighted in bold) and closely related species.

|  |  |  | 1 | 2 | 3 | 4 | 5 | 6 | 7 | 8 | 9 | 10 | 11 | 12 |
| --- | --- | --- | --- | --- | --- | --- | --- | --- | --- | --- | --- | --- | --- | --- |
| 1 | IB R.6455 | *S. tamchucensis* sp. nov. | - |  |  |  |  |  |  |  |  |  |  |  |
| 2 | IB R.6458 | *S. tamchucensis* sp. nov. | 1.39 | - |  |  |  |  |  |  |  |  |  |  |
| 3 | OM420325 | *S. tonkinensiis* | 19.08 | 19.39 | - |  |  |  |  |  |  |  |  |  |
| 4 | OM420326 | *S. tonkinensis* | 18.77 | 19.08 | 0.77 | - |  |  |  |  |  |  |  |  |
| 5 | OM420415 | *S. sp.* | 17.54 | 17.69 | 8.15 | 8.00 | - |  |  |  |  |  |  |  |
| 6 | OM420414 | *S. sp.* | 17.54 | 17.69 | 8.31 | 8.15 | 0.46 | - |  |  |  |  |  |  |
| 7 | MH237969 | *S. buenloicus* | 21.54 | 20.62 | 20.31 | 20.15 | 19.69 | 19.54 | - |  |  |  |  |  |
| 8 | MH237970 | *S. buenloicus* | 21.54 | 20.62 | 20.00 | 19.85 | 19.39 | 19.23 | 0.31 | - |  |  |  |  |
| 9 | MH237971 | *S. yersinisi* | 18.46 | 18.31 | 19.23 | 18.77 | 18.31 | 18.46 | 16.62 | 16.31 | - |  |  |  |
| 10 | MH237972 | *S. yersinisi* | 18.46 | 18.31 | 19.23 | 18.77 | 18.31 | 18.46 | 16.62 | 16.31 | 0.15 | - |  |  |
| 11 | MH274672 | *S. scutatus* | 18.62 | 18.77 | 20.00 | 19.69 | 18.46 | 18.77 | 20.46 | 20.46 | 18.92 | 19.08 | - |  |
| 12 | MH274671 | *S. scutatus* | 18.77 | 18.92 | 19.39 | 19.08 | 17.85 | 18.15 | 20.46 | 20.46 | 18.77 | 18.92 | 2.92 | - |
| 13 | MH274678 | *S. solomonis* | 17.85 | 18.00 | 20.15 | 19.85 | 18.92 | 19.23 | 20.00 | 20.00 | 18.00 | 17.85 | 7.08 | 5.85 |
| 14 | MH274679 | *S. solomonis* | 18.15 | 18.31 | 20.15 | 19.85 | 18.62 | 18.92 | 20.00 | 20.00 | 18.00 | 17.85 | 6.77 | 5.54 |
| 15 | OP942215 | *S. cryptotis* | 19.54 | 19.39 | 20.92 | 20.62 | 19.69 | 19.69 | 19.54 | 19.54 | 18.77 | 18.77 | 20.00 | 19.69 |
| 16 | MK450438 | *S. indicus* | 18.92 | 18.92 | 20.00 | 19.69 | 19.23 | 19.23 | 20.77 | 20.77 | 18.62 | 18.46 | 19.23 | 19.08 |
| 17 | OM117611 | *S. indicus* | 19.39 | 19.23 | 20.62 | 20.31 | 20.00 | 20.00 | 21.39 | 21.39 | 18.92 | 18.77 | 19.54 | 19.39 |
| 18 | MH329292 | *S. incognitus* | 19.23 | 19.69 | 21.39 | 20.92 | 19.69 | 19.54 | 22.62 | 22.31 | 19.69 | 19.69 | 20.62 | 21.23 |
| 19 | NC 04112 | *S. incognitus* | 19.23 | 19.69 | 21.39 | 20.92 | 19.69 | 19.54 | 22.62 | 22.31 | 19.69 | 19.69 | 20.62 | 21.23 |
| 20 | MH119629 | *S. maculatus* | 21.39 | 21.23 | 23.08 | 22.62 | 20.77 | 20.77 | 23.08 | 23.08 | 20.00 | 20.15 | 20.00 | 20.15 |
| 21 | MG935700 | *S. maculatus* | 22.00 | 21.85 | 21.85 | 21.23 | 20.46 | 20.46 | 22.46 | 22.46 | 20.00 | 20.15 | 20.62 | 21.08 |
| 22 | MK089440 | *S. dussumier* | 21.43 | 21.61 | 20.55 | 20.14 | 18.84 | 18.86 | 23.08 | 22.71 | 20.25 | 20.45 | 21.37 | 21.55 |
| 23 | PP931018 | *S. veunsaiense* | 20.36 | 20.21 | 22.12 | 21.80 | 21.00 | 21.16 | 20.36 | 20.36 | 19.57 | 19.57 | 19.56 | 19.40 |
| 24 | PP931016 | *S. veunsaiense* | 20.36 | 20.36 | 21.64 | 21.32 | 20.52 | 20.68 | 19.88 | 19.88 | 19.41 | 19.41 | 19.40 | 19.24 |
| 25 | KJ643142 | *Plestiodon elegans* | 22.15 | 22.15 | 22.77 | 22.46 | 21.08 | 21.08 | 23.08 | 22.77 | 20.62 | 20.77 | 21.08 | 21.08 |

Uncorrected ("p") distance matrix (continued)

|  |  |  | 13 | 14 | 15 | 16 | 17 | 18 | 19 | 20 | 21 | 22 | 23 | 24 | 25 |
| --- | --- | --- | --- | --- | --- | --- | --- | --- | --- | --- | --- | --- | --- | --- | --- |
| 13 | MH274678 | *S. solomonis* | - |  |  |  |  |  |  |  |  |  |  |  |  |
| 14 | MH274679 | *S. solomonis* | 0.31 | - |  |  |  |  |  |  |  |  |  |  |  |
| 15 | OP942215 | *S. cryptotis* | 19.39 | 19.39 | - |  |  |  |  |  |  |  |  |  |  |
| 16 | MK450438 | *S. indicus* | 20.00 | 19.69 | 20.31 | - |  |  |  |  |  |  |  |  |  |
| 17 | OM117611 | *S. indicus* | 20.00 | 19.69 | 20.62 | 1.85 | - |  |  |  |  |  |  |  |  |
| 18 | MH329292 | *S. incognitus* | 20.77 | 20.46 | 20.77 | 20.77 | 20.15 | - |  |  |  |  |  |  |  |
| 19 | NC 04112 | *S. incognitus* | 20.77 | 20.46 | 20.77 | 20.77 | 20.15 | 0.00 | - |  |  |  |  |  |  |
| 20 | MH119629 | *S. maculatus* | 19.39 | 19.39 | 19.39 | 22.00 | 22.46 | 22.15 | 22.15 | - |  |  |  |  |  |
| 21 | MG935700 | *S. maculatus* | 20.00 | 20.00 | 18.77 | 23.23 | 23.08 | 21.85 | 21.85 | 6.00 | - |  |  |  |  |
| 22 | MK089440 | *S. dussumier* | 21.27 | 20.89 | 20.08 | 21.69 | 21.88 | 21.83 | 21.83 | 13.01 | 14.12 | - |  |  |  |
| 23 | PP931018 | *S. veunsaiense* | 19.41 | 19.41 | 19.74 | 19.40 | 19.88 | 19.92 | 19.92 | 23.43 | 22.94 | 22.21 | - |  |  |
| 24 | PP931016 | *S. veunsaiense* | 19.25 | 19.25 | 19.57 | 19.24 | 20.04 | 19.76 | 19.76 | 23.26 | 22.78 | 21.84 | 0.48 | - |  |
| 25 | KJ643142 | *Plestiodon elegans* | 21.08 | 20.77 | 22.46 | 22.00 | 22.46 | 23.39 | 23.39 | 22.15 | 22.62 | 19.25 | 20.66 | 20.82 | - |

**Table S3**. Significant p-values from the results of the ANOVA and TukeyHSD analyses comparing the size-corrected morphometric of *Sphenomorphus tamchucensis* sp. nov. to closely related species.

| Morphometric characters | SVL | AG | SL | STL | SFlL | END | EL | HL | HW | HH | TYD | FlL | HlL |
| --- | --- | --- | --- | --- | --- | --- | --- | --- | --- | --- | --- | --- | --- |
| *S. tamchucensis-**S. buenloicus* | 0.009 | <0.001 | <0.001 | <0.001 | <0.001 | <0.001 | <0.001 | <0.001 | <0.001 | <0.001 | <0.001 | <0.001 | <0.001 |
| *S. tamchucensis-**S. cryptotis* | <0.001 | <0.001 | <0.001 | <0.001 | <0.001 | <0.001 | <0.001 | <0.001 | <0.001 | <0.001 | <0.001 | <0.001 | <0.001 |
| *S. tamchucensis-**S. incognitus* | <0.001 | <0.001 | <0.001 | <0.001 | <0.001 | <0.001 | <0.001 | <0.001 | <0.001 | <0.001 | <0.001 | <0.001 | <0.001 |
| *S. tamchucensis-S. indicus* | <0.001 | <0.001 | <0.001 | <0.001 | <0.001 | <0.001 | <0.001 | <0.001 | <0.001 | <0.001 | <0.001 | <0.001 | <0.001 |
| *S. tamchucensis-S. lineopunctulatus* |  | <0.001 | <0.001 | <0.001 | <0.001 | <0.001 | <0.001 | <0.001 | <0.001 | <0.001 | <0.001 | <0.001 | <0.001 |
| *S. tonkinensis-S. tamchucensis* |  | 0.036 | 0.001 |  |  | <0.001 | <0.001 |  |  |  |  | <0.001 | <0.001 |

**Table S4**. Significant p-values from the results of the ANOVA and TukeyHSD analyses comparing the meristic characters of *Sphenomorphus tamchucensis* sp. nov. to closely related species.

| Morphometric characters | So | Nu | PC | PS | SC | PT | SL | IF | MB | PV | VR | FL4 | TL4 |
| --- | --- | --- | --- | --- | --- | --- | --- | --- | --- | --- | --- | --- | --- |
| *S. tamchucensis-S. buenloicus* |  |  | <0.001 |  |  | <0.001 |  |  | <0.001 |  |  | <0.001 | <0.001 |
| *S. tamchucensis-S. cryptotis* | <0.001 | <0.001 | <0.001 | <0.001 | <0.001 | <0.001 |  |  | <0.001 | <0.001 | <0.001 | <0.001 | <0.001 |
| *S. tamchucensis-S. incognitus* |  |  | <0.001 |  | <0.001 |  |  | 0.004 | <0.001 | <0.001 | <0.001 | <0.001 | <0.001 |
| *S. tamchucensis-**S. indicus* |  | <0.001 |  | <0.001 | 0.007 | <0.001 |  | <0.001 | <0.001 | <0.001 | <0.001 | <0.001 | <0.001 |
| *S. tamchucensis-**S. lineopunctulatus* |  |  | <0.001 |  | <0.001 |  |  |  | <0.001 | <0.001 | <0.001 | 0.005 | <0.001 |
| *S. tonkinensis-S. tamchucensis* |  |  |  | <0.001 | <0.001 |  |  |  | <0.001 | <0.001 | <0.001 | 0.003 | 0.011 |

**Table S5.** Summary statistics of size-corrected morphometric characters among the new *Sphenomorphus* population and their closely related species

| Species | SVL | AG | SL | STL | SFlL | END | EL | HL | HW | HH | TYD | FlL | HlL |
| --- | --- | --- | --- | --- | --- | --- | --- | --- | --- | --- | --- | --- | --- |
| *S. buenloicus* (N=11) | | | | | | | | | | | | | |
| Mean | 1.71 | 1.42 | 0.63 | 0.99 | 1.26 | 0.40 | 0.47 | 0.97 | 0.82 | 0.72 | 0.15 | 1.13 | 1.29 |
| SD | 0.0369 | 0.0114 | 0.0223 | 0.0126 | 0.0180 | 0.0498 | 0.0218 | 0.0182 | 0.0179 | 0.0404 | 0.0374 | 0.0381 | 0.0277 |
| Lower | 1.67 | 1.40 | 0.58 | 0.97 | 1.23 | 0.27 | 0.43 | 0.94 | 0.80 | 0.64 | 0.11 | 1.05 | 1.23 |
| Upper | 1.76 | 1.44 | 0.66 | 1.01 | 1.30 | 0.46 | 0.50 | 1.00 | 0.85 | 0.78 | 0.22 | 1.21 | 1.33 |
| *S. cryptotis* (N=17) | | | | | | | | | | | | | |
| Mean | 1.85 | 1.56 | 0.78 | 1.15 | 1.40 | 0.61 | 0.65 | 1.14 | 0.94 | 0.88 | 0.28 | 1.30 | 1.47 |
| SD | 0.0387 | 0.0113 | 0.0230 | 0.0160 | 0.0135 | 0.0188 | 0.0372 | 0.0139 | 0.0229 | 0.0264 | 0.0507 | 0.0137 | 0.0174 |
| Lower | 1.78 | 1.54 | 0.73 | 1.12 | 1.36 | 0.57 | 0.60 | 1.11 | 0.90 | 0.82 | 0.17 | 1.27 | 1.44 |
| Upper | 1.90 | 1.58 | 0.81 | 1.18 | 1.42 | 0.64 | 0.72 | 1.16 | 0.97 | 0.93 | 0.35 | 1.33 | 1.49 |
| *S. incognitus* (N=10) | | | | | | | | | | | | | |
| Mean | 1.85 | 1.57 | 0.81 | 1.16 | 1.43 | 0.63 | 0.66 | 1.14 | 1.01 | 0.94 | 0.37 | 1.32 | 1.51 |
| SD | 0.1482 | 0.0161 | 0.0252 | 0.0064 | 0.0082 | 0.0351 | 0.0212 | 0.0115 | 0.0146 | 0.0214 | 0.0134 | 0.0121 | 0.0202 |
| Lower | 1.64 | 1.53 | 0.77 | 1.15 | 1.42 | 0.59 | 0.64 | 1.11 | 0.98 | 0.91 | 0.35 | 1.30 | 1.48 |
| Upper | 2.01 | 1.59 | 0.85 | 1.17 | 1.45 | 0.69 | 0.71 | 1.15 | 1.03 | 0.98 | 0.40 | 1.34 | 1.54 |
| *S. indicus* (N=11) | | | | | | | | | | | | | |
| Mean | 1.86 | 1.59 | 0.75 | 1.14 | 1.42 | 0.59 | 0.60 | 1.10 | 1.00 | 0.92 | 0.23 | 1.30 | 1.49 |
| SD | 0.0502 | 0.0171 | 0.0196 | 0.0136 | 0.0186 | 0.0240 | 0.0250 | 0.0216 | 0.0138 | 0.0395 | 0.0620 | 0.0183 | 0.0247 |
| Lower | 1.77 | 1.57 | 0.71 | 1.12 | 1.39 | 0.56 | 0.54 | 1.07 | 0.99 | 0.86 | 0.16 | 1.26 | 1.43 |
| Upper | 1.95 | 1.61 | 0.77 | 1.17 | 1.46 | 0.62 | 0.62 | 1.14 | 1.03 | 0.99 | 0.35 | 1.32 | 1.52 |
| *S. lineopunctulatus* (N=3) | | | | | | | | | | | | | |
| Mean | 1.71 | 1.41 | 0.71 | 1.05 | 1.31 | 0.46 | 0.55 | 1.04 | 0.88 | 0.76 | 0.19 | 1.19 | 1.40 |
| SD | 0.1468 | 0.0155 | 0.0024 | 0.0147 | 0.0023 | 0.0073 | 0.0018 | 0.0067 | 0.0236 | 0.0334 | 0.0383 | 0.0145 | 0.0041 |
| Lower | 1.55 | 1.39 | 0.71 | 1.04 | 1.30 | 0.45 | 0.55 | 1.03 | 0.86 | 0.73 | 0.15 | 1.17 | 1.39 |
| Upper | 1.83 | 1.42 | 0.71 | 1.07 | 1.31 | 0.46 | 0.55 | 1.04 | 0.90 | 0.80 | 0.23 | 1.20 | 1.40 |
| *S. tamchucensis* (N=11) | | | | | | | | | | | | | |
| Mean | 1.59 | 1.33 | 0.46 | 0.88 | 1.12 | 0.05 | 0.32 | 0.86 | 0.67 | 0.58 | 0.03 | 0.93 | 1.11 |
| SD | 0.0210 | 0.0143 | 0.0172 | 0.0120 | 0.0130 | 0.0364 | 0.0102 | 0.0072 | 0.0426 | 0.0222 | 0.0129 | 0.0163 | 0.0120 |
| Lower | 1.56 | 1.30 | 0.43 | 0.86 | 1.10 | -0.01 | 0.29 | 0.85 | 0.56 | 0.53 | 0.01 | 0.91 | 1.09 |
| Upper | 1.62 | 1.36 | 0.49 | 0.90 | 1.15 | 0.11 | 0.33 | 0.87 | 0.71 | 0.60 | 0.05 | 0.95 | 1.14 |
| *S. tonkinensis* (N=11) | | | | | | | | | | | | | |
| Mean | 1.59 | 1.31 | 0.51 | 0.88 | 1.13 | 0.26 | 0.39 | 0.87 | 0.70 | 0.61 | 0.07 | 1.00 | 1.18 |
| SD | 0.0777 | 0.0124 | 0.0340 | 0.0213 | 0.0208 | 0.0568 | 0.0343 | 0.0150 | 0.0354 | 0.0463 | 0.0421 | 0.0151 | 0.0149 |
| Lower | 1.44 | 1.29 | 0.45 | 0.86 | 1.10 | 0.20 | 0.31 | 0.85 | 0.67 | 0.52 | 0.00 | 0.97 | 1.15 |
| Upper | 1.69 | 1.33 | 0.57 | 0.92 | 1.16 | 0.38 | 0.42 | 0.90 | 0.78 | 0.67 | 0.13 | 1.03 | 1.20 |

**Table S6.** Summary statistics of meristic characters among the new *Sphenomorphus* population and their closely related species

| Species | SO | Nu | L | PR | PRS | SC | PT | ST | SL | IF | Cs | TMR | Mds | VR | PrC | FL4 | TL4 |
| --- | --- | --- | --- | --- | --- | --- | --- | --- | --- | --- | --- | --- | --- | --- | --- | --- | --- |
| *S. buenloicus* (N=11) | | | | | | | | | | | | | | | | | |
| Mean | 4.00 | 0.455 | 2.00 | 1.00 | 2.000 | 7.091 | 1.00 | 2.00 | 6.864 | 6.000 | 3.00 | 33.09 | 63.18 | 59.36 | 2.00 | 11.545 | 18.182 |
| SD | 0 | 0.5681 | 0 | 0 | 0 | 0.3015 | 0 | 0 | 0.3233 | 0 | 0 | 1.044 | 2.359 | 1.963 | 0 | 0.4719 | 1.1461 |
| Lower | 4 | 0.0 | 2 | 1 | 2.0 | 7.0 | 1 | 2 | 6.0 | 6.0 | 3 | 32 | 61 | 57 | 2 | 11.0 | 16.0 |
| Upper | 4 | 1.5 | 2 | 1 | 2.0 | 8.0 | 1 | 2 | 7.0 | 6.0 | 3 | 34 | 69 | 63 | 2 | 12.0 | 20.0 |
| *S. cryptotis* (N=17) | | | | | | | | | | | | | | | | | |
| Mean | 2.00 | 1.147 | 2.00 | 1.00 | 1.000 | 9.912 | 1.00 | 2.00 | 7.118 | 6.206 | 3.00 | 35.65 | 77.59 | 79.82 | 2.00 | 13.382 | 18.882 |
| SD | 0 | 0.4926 | 0 | 0 | 0 | 0.7339 | 0 | 0 | 0.2811 | 0.3561 | 0 | 1.766 | 5.269 | 4.475 | 0 | 0.9442 | 1.3054 |
| Lower | 2 | 0.5 | 2 | 1 | 1.0 | 8.5 | 1 | 2 | 7.0 | 6.0 | 3 | 32 | 69 | 74 | 2 | 11.0 | 16.5 |
| Upper | 2 | 2.0 | 2 | 1 | 1.0 | 11.0 | 1 | 2 | 8.0 | 7.0 | 3 | 38 | 88 | 93 | 2 | 15.0 | 22.0 |
| *S. incognitus* (N=10) | | | | | | | | | | | | | | | | | |
| Mean | 4.00 | 0.600 | 2.00 | 1.00 | 1.400 | 9.150 | 2.00 | 2.00 | 7.000 | 6.450 | 3.00 | 37.80 | 73.90 | 76.60 | 2.00 | 12.700 | 20.400 |
| SD | 0 | 0.9661 | 0 | 0 | 0.5164 | 0.9733 | 0 | 0 | 0 | 0.4972 | 0 | 0.632 | 2.885 | 3.596 | 0 | 0.6325 | 1.4298 |
| Lower | 4 | 0.0 | 2 | 1 | 1.0 | 8.0 | 2 | 2 | 7.0 | 6.0 | 3 | 36 | 70 | 73 | 2 | 12.0 | 18.0 |
| Upper | 4 | 2.0 | 2 | 1 | 2.0 | 11.0 | 2 | 2 | 7.0 | 7.0 | 3 | 38 | 80 | 84 | 2 | 14.0 | 23.0 |
| *S. indicus* (N=11) | | | | | | | | | | | | | | | | | |
| Mean | 4.00 | 2.000 | 2.00 | 2.00 | 2.909 | 8.273 | 1.18 | 2.00 | 7.000 | 7.000 | 3.00 | 34.73 | 69.64 | 66.91 | 2.00 | 10.909 | 17.318 |
| SD | 0 | 1.0954 | 0 | 0 | 0.3015 | 0.6467 | 0.405 | 0 | 0 | 0 | 0 | 1.348 | 3.325 | 2.427 | 0 | 0.9439 | 1.4190 |
| Lower | 4 | 1.0 | 2 | 2 | 2.0 | 7.0 | 1 | 2 | 7.0 | 7.0 | 3 | 32 | 64 | 62 | 2 | 10.0 | 16.0 |
| Upper | 4 | 4.0 | 2 | 2 | 3.0 | 9.0 | 2 | 2 | 7.0 | 7.0 | 3 | 36 | 75 | 70 | 2 | 13.0 | 20.0 |
| *S. lineopunctulatus* (N=3) | | | | | | | | | | | | | | | | | |
| Mean | 4.00 | 0.333 | 2.00 | 1.00 | 2.000 | 9.167 | 2.00 | 2.00 | 7.000 | 6.167 | 3.00 | 38.67 | 75.00 | 82.00 | 2.00 | 11.333 | 19.833 |
| SD | 0 | 0.5774 | 0 | 0 | 0 | 0.2887 | 0 | 0 | 0 | 0.2887 | 0 | 1.155 | 1.000 | 3.606 | 0 | 0.5774 | 0.5774 |
| Lower | 4 | 0.0 | 2 | 1 | 2.0 | 9.0 | 2 | 2 | 7.0 | 6.0 | 3 | 38 | 74 | 79 | 2 | 11.0 | 19.5 |
| Upper | 4 | 1.0 | 2 | 1 | 2.0 | 9.5 | 2 | 2 | 7.0 | 6.5 | 3 | 40 | 76 | 86 | 2 | 12.0 | 20.5 |
| *S. tamchucensis* (N=11) | | | | | | | | | | | | | | | | | |
| Mean | 4.00 | 0.000 | 2.00 | 2.00 | 1.682 | 7.273 | 2.00 | 2.00 | 7.000 | 6.000 | 3.00 | 28.00 | 61.73 | 57.64 | 2.00 | 9.545 | 14.364 |
| SD | 0 | 0 | 0 | 0 | 0.4622 | 0.4101 | 0 | 0 | 0 | 0 | 0 | 0 | 1.489 | 1.804 | 0 | 0.5681 | 0.8090 |
| Lower | 4 | 0.0 | 2 | 2 | 1.0 | 7.0 | 2 | 2 | 7.0 | 6.0 | 3 | 28 | 58 | 56 | 2 | 8.5 | 13.0 |
| Upper | 4 | 0.0 | 2 | 2 | 2.0 | 8.0 | 2 | 2 | 7.0 | 6.0 | 3 | 28 | 63 | 61 | 2 | 10.0 | 15.0 |
| *S. tonkinensis* (N=11) | | | | | | | | | | | | | | | | | |
| Mean | 4.00 | 0.227 | 2.00 | 2.00 | 3.000 | 8.727 | 2.00 | 2.00 | 7.000 | 6.045 | 3.00 | 33.27 | 69.45 | 63.73 | 2.00 | 10.773 | 16.227 |
| SD | 0 | 0.3438 | 0 | 0 | 0 | 0.4671 | 0 | 0 | 0 | 0.1508 | 0 | 1.009 | 2.979 | 3.552 | 0 | 0.3438 | 1.1481 |
| Lower | 4 | 0.0 | 2 | 2 | 3.0 | 8.0 | 2 | 2 | 7.0 | 6.0 | 3 | 32 | 65 | 58 | 2 | 10.0 | 15.0 |
| Upper | 4 | 1.0 | 2 | 2 | 3.0 | 9.0 | 2 | 2 | 7.0 | 6.5 | 3 | 34 | 72 | 71 | 2 | 11.0 | 18.5 |

**Table S7.** Summary statistics of the principal component analysis for the *Sphenomorphus* species group

|  | PC1 | PC2 | PC3 | PC4 | PC5 | PC6 | PC7 | PC8 | PC9 | PC10 | PC11 | PC12 | PC13 |
| --- | --- | --- | --- | --- | --- | --- | --- | --- | --- | --- | --- | --- | --- |
| Standard deviation | 4.112004 | 1.618831 | 1.23028 | 1.174488 | 0.937995 | 0.825174 | 0.659125 | 0.60904 | 0.549818 | 0.458776 | 0.387975 | 0.34055 | 0.323487 |
| Proportion of Variance | 0.65033 | 0.10079 | 0.05821 | 0.05305 | 0.03384 | 0.02619 | 0.01671 | 0.01427 | 0.01163 | 0.0081 | 0.00579 | 0.00446 | 0.00402 |
| Cumulative Proportion | 0.65033 | 0.75112 | 0.80934 | 0.86239 | 0.89623 | 0.92242 | 0.93913 | 0.9534 | 0.96502 | 0.97312 | 0.97891 | 0.98337 | 0.98739 |
| Eigenvalues | 16.90857 | 2.620614 | 1.513589 | 1.379423 | 0.879835 | 0.680912 | 0.434446 | 0.370929 | 0.3023 | 0.210475 | 0.150525 | 0.115974 | 0.104644 |
| SVL | -0.208 | -0.09755 | 0.040382 | -0.08849 | 0.038655 | -0.31805 | 0.310237 | -0.13649 | 0.26431 | -0.25601 | 0.65554 | -0.15876 | -0.24655 |
| AG | -0.22798 | -0.12703 | -0.00768 | -0.10867 | 0.119309 | -0.11302 | -0.0737 | 0.177187 | -0.1194 | 0.009155 | 0.025336 | -0.13466 | 0.151067 |
| SL | -0.23845 | -0.03921 | -0.06615 | 0.027639 | 0.022493 | 0.010597 | -0.12113 | 0.013056 | 0.028032 | 0.064367 | -0.01882 | 0.01032 | -0.05382 |
| STL | -0.2385 | -0.06929 | -0.03385 | -0.03013 | 0.076582 | -0.03572 | -0.1184 | 0.036187 | -0.07769 | 0.036727 | -0.01113 | -0.11835 | -0.05004 |
| SFlL | -0.23501 | -0.11944 | -0.06227 | -0.02551 | 0.067155 | -0.03958 | -0.11539 | 0.061187 | -0.01764 | -0.00933 | -0.01853 | -0.09546 | 0.073648 |
| END | -0.23462 | -0.07023 | -0.049 | 0.026434 | -0.07113 | 0.156178 | 0.002698 | 0.097489 | 0.060132 | 0.028489 | 0.021675 | 0.073801 | 0.031406 |
| EL | -0.23818 | -0.00358 | 0.003207 | 0.043549 | -0.00683 | 0.005758 | -0.0728 | 0.106024 | 0.019344 | 0.03128 | -0.07687 | 0.019771 | 0.189962 |
| HL | -0.24042 | -0.02641 | -0.02734 | -0.03063 | 0.051715 | -0.02681 | -0.10289 | 0.079632 | -0.0578 | 0.056592 | -0.04448 | -0.05399 | -0.07648 |
| HW | -0.22876 | -0.16821 | -0.05526 | 0.043784 | 0.061397 | 0.010764 | -0.12287 | 0.046913 | -0.00355 | 0.01087 | 0.028247 | -0.08235 | -0.01369 |
| HH | -0.23057 | -0.13653 | -0.02522 | 0.031933 | 0.121563 | -0.01261 | -0.04175 | 0.133391 | -0.07853 | 0.030755 | 0.029895 | -0.11082 | 0.269332 |
| TYD | -0.22144 | -0.00443 | -0.09033 | 0.10909 | 0.078454 | -0.07692 | -0.3181 | -0.11622 | -0.08581 | 0.014151 | 0.162937 | 0.738145 | -0.27784 |
| FlL | -0.23847 | -0.08262 | -0.05038 | -0.00941 | -0.00782 | 0.04294 | -0.03791 | 0.071891 | -0.00546 | 0.063492 | -0.01749 | -0.06302 | -0.03352 |
| HlL | -0.23717 | -0.10589 | -0.02239 | 0.032477 | 0.000938 | 0.022696 | -0.06997 | 0.051002 | -0.02246 | 0.028962 | -0.06103 | -0.02283 | -0.09872 |
| Supraoculars | 0.134441 | -0.35348 | -0.17714 | 0.417926 | 0.087207 | 0.028929 | -0.07272 | -0.2547 | 0.225799 | -0.11895 | 0.174695 | 0.026674 | 0.144943 |
| Nuchals | -0.12549 | -0.2798 | 0.176805 | -0.33106 | -0.3578 | 0.024045 | -0.16207 | -0.70348 | -0.23642 | -0.01784 | -0.07499 | -0.06758 | 0.149449 |
| Preocular | 0.153876 | -0.32545 | 0.381275 | -0.01069 | -0.0152 | -0.064 | 0.011469 | 0.23414 | -0.41929 | 0.021487 | 0.174879 | -0.18243 | -0.19673 |
| Presuboculars | 0.102652 | -0.41039 | 0.116411 | 0.156907 | -0.29134 | 0.557939 | 0.043293 | 0.228458 | -0.04081 | -0.10528 | 0.100041 | 0.145157 | -0.20887 |
| Supraciliaries | -0.15482 | 0.211321 | 0.437956 | 0.094487 | -0.25576 | -0.08025 | 0.196912 | -0.05904 | 0.108344 | 0.576395 | 0.152617 | 0.18646 | 0.048246 |
| Primary.temporals | 0.117791 | 0.037271 | 0.177729 | 0.642235 | 0.126405 | -0.29836 | -0.20318 | -0.11955 | -0.33979 | 0.085187 | 0.053459 | -0.13282 | 0.088264 |
| Supralabials..R.L. | -0.04961 | 0.137348 | 0.483752 | -0.07172 | 0.642557 | 0.466891 | -0.13954 | -0.22219 | 0.151935 | -0.0701 | 0.06457 | -0.05391 | 0.007689 |
| Infralabials | -0.11347 | -0.41631 | 0.220944 | 0.043085 | 0.253008 | -0.20346 | 0.530965 | 0.022324 | 0.094225 | 0.06956 | -0.4082 | 0.257381 | 0.046806 |
| Midbody.scale.rows | -0.19635 | 0.019246 | 0.008096 | 0.342089 | -0.26346 | 0.220867 | -0.02389 | -0.04529 | 0.388391 | 0.213288 | -0.01578 | -0.31844 | 0.018812 |
| Paravertebral.scales | -0.17704 | 0.191224 | 0.337437 | 0.117438 | -0.26398 | -0.04292 | 0.00425 | 0.176579 | 0.017215 | -0.61367 | 0.028218 | 0.201687 | 0.462561 |
| Ventrals.in.transverse.rows | -0.19803 | 0.219054 | 0.215293 | 0.142817 | -0.13358 | -0.10458 | -0.06345 | -0.0259 | 0.014058 | -0.29575 | -0.36518 | -0.17953 | -0.58843 |
| Lamellae.on.finger.IV | -0.18773 | 0.267595 | -0.12331 | 0.006353 | 0.025745 | 0.279155 | 0.279033 | 0.045626 | -0.4616 | 0.086308 | 0.307634 | -0.00274 | 0.042878 |
| Lamellae.on.toe.IV | -0.18162 | 0.103861 | -0.25999 | 0.264349 | 0.064973 | 0.195668 | 0.467518 | -0.32557 | -0.28182 | -0.15943 | -0.15955 | -0.02754 | -0.03753 |

**Table S7 (Continued)**

|  | PC14 | PC15 | PC16 | PC17 | PC18 | PC19 | PC20 | PC21 | PC22 | PC23 | PC24 | PC25 | PC26 |
| --- | --- | --- | --- | --- | --- | --- | --- | --- | --- | --- | --- | --- | --- |
| Standard deviation | 0.270315 | 0.240188 | 0.200296 | 0.190398 | 0.166662 | 0.153563 | 0.142794 | 0.135296 | 0.108278 | 0.092379 | 0.070636 | 0.057073 | 0.045535 |
| Proportion of Variance | 0.00281 | 0.00222 | 0.00154 | 0.00139 | 0.00107 | 0.00091 | 0.00078 | 7.00E-04 | 0.00045 | 0.00033 | 0.00019 | 0.00013 | 8.00E-05 |
| Cumulative Proportion | 0.9902 | 0.99242 | 0.99397 | 0.99536 | 0.99643 | 0.99734 | 0.99812 | 0.99882 | 0.99927 | 0.9996 | 0.99979 | 0.99992 | 1 |
| Eigenvalues | 0.07307 | 0.05769 | 0.040119 | 0.036251 | 0.027776 | 0.023582 | 0.02039 | 0.018305 | 0.011724 | 0.008534 | 0.004989 | 0.003257 | 0.002073 |
| SVL | -0.01838 | 0.094619 | 0.153361 | 0.080284 | -0.14158 | -0.04973 | -0.04805 | -0.05589 | -0.02807 | -0.01981 | -0.00571 | -0.01958 | -0.01752 |
| AG | 0.171266 | -0.18815 | 0.151704 | 0.14224 | -0.04928 | -0.11519 | 0.515981 | 0.576409 | -0.17922 | 0.150312 | 0.069043 | -0.07426 | 0.114406 |
| SL | 0.148872 | -0.03339 | 0.167316 | -0.19246 | -0.0042 | 0.619999 | -0.02408 | -0.20094 | -0.61394 | 0.027721 | -0.13354 | -0.02045 | 0.055559 |
| STL | -0.00397 | 0.005976 | -0.17148 | -0.13944 | -0.11034 | 0.098259 | -0.04646 | -0.03917 | 0.127157 | 0.45957 | 0.317281 | 0.013391 | -0.69795 |
| SFlL | 0.096625 | 0.030723 | -0.18493 | 0.112283 | -0.03367 | 0.011757 | -0.06748 | -0.12856 | 0.307263 | 0.361422 | -0.72308 | -0.19882 | 0.143943 |
| END | -0.0345 | 0.058629 | 0.556606 | -0.02105 | 0.518706 | 0.017203 | 0.306016 | -0.293 | 0.332859 | -0.01701 | 0.053851 | -0.04038 | -0.08533 |
| EL | -0.15804 | -0.06164 | 0.223518 | 0.648316 | 0.111911 | -0.04451 | -0.55161 | 0.123001 | -0.16301 | -0.03541 | 0.035112 | 0.030061 | -0.13443 |
| HL | -0.00117 | 0.09132 | -0.07404 | 0.045617 | -0.09364 | -0.02049 | -0.12408 | -0.24919 | 0.114953 | 0.311702 | 0.512335 | 0.098394 | 0.645819 |
| HW | 0.043983 | -0.25962 | 0.169043 | -0.47804 | -0.0658 | 0.112612 | -0.40401 | 0.366612 | 0.359831 | -0.29639 | -0.01729 | 0.136247 | 0.089802 |
| HH | 0.082375 | 0.069673 | -0.08512 | -0.32772 | 0.083043 | -0.63369 | -0.10481 | -0.28785 | -0.35989 | -0.17682 | -0.03037 | -0.07285 | -0.06036 |
| TYD | -0.16674 | 0.200124 | -0.09979 | -0.04419 | 0.063476 | -0.1375 | 0.04015 | 0.190955 | -0.06287 | 0.012147 | -0.05495 | -0.00724 | 0.003356 |
| FlL | 0.158979 | 0.136585 | -0.22975 | 0.22459 | -0.09036 | 0.044249 | 0.230618 | -0.11231 | 0.070434 | -0.30538 | -0.11444 | 0.757636 | -0.08546 |
| HlL | 0.119256 | -0.00114 | -0.27823 | 0.22524 | -0.15692 | 0.161383 | 0.126665 | -0.12342 | 0.159927 | -0.53453 | 0.194268 | -0.57543 | -0.05622 |
| Supraoculars | 0.097662 | -0.44914 | -0.29726 | 0.102605 | 0.333463 | 0.040319 | 0.041156 | -0.05267 | -0.02893 | 0.087993 | 0.115666 | 0.058995 | 0.045521 |
| Nuchals | -0.11687 | -0.03183 | 0.063185 | 0.010583 | -0.04737 | -0.03456 | 0.035721 | -0.03884 | -0.02328 | -0.02367 | 0.000201 | 0.011186 | 0.019885 |
| Preocular | -0.05543 | 0.243152 | -0.20732 | -0.0002 | 0.47333 | 0.157073 | -0.10762 | 0.129399 | -0.05068 | -0.02768 | -0.00308 | -0.00341 | 0.035186 |
| Presuboculars | 0.151311 | -0.05758 | 0.170728 | 0.056365 | -0.37976 | -0.13717 | -0.03045 | -0.03781 | -0.06354 | 0.125776 | -0.02457 | -0.01306 | -0.02487 |
| Supraciliaries | 0.392375 | -0.17924 | -0.08181 | -0.01516 | 0.065589 | -0.0506 | -0.06007 | 0.01917 | 0.046103 | 0.076516 | 0.007524 | -0.01584 | -0.00636 |
| Primary.temporals | -0.05301 | 0.072153 | 0.300497 | 0.054175 | -0.29909 | 0.007166 | 0.09371 | -0.13839 | 0.082798 | -0.03516 | -0.03553 | 0.023059 | -0.02292 |
| Supralabials..R.L. | 0.024074 | 0.0403 | 0.017306 | 0.034366 | -0.00158 | 0.011123 | 0.016852 | 0.013604 | 0.01025 | -0.01416 | -0.00408 | 0.001692 | 0.008412 |
| Infralabials | -0.30706 | -0.06174 | 0.001313 | -0.05238 | -0.11495 | 0.060152 | 0.067451 | -0.04573 | 0.000372 | 0.018979 | -0.02711 | 0.032809 | 0.006413 |
| Midbody.scale.rows | -0.44842 | 0.319332 | -0.14157 | -0.10269 | 0.012379 | 0.002808 | 0.109652 | 0.252378 | -0.08041 | 0.032114 | -0.02871 | -0.05565 | 0.062349 |
| Paravertebral.scales | 0.010969 | 0.058459 | -0.14378 | -0.104 | -0.01559 | 0.159067 | 0.032227 | 0.015986 | 0.052373 | 0.010649 | 0.058313 | 0.028108 | 0.021682 |
| Ventrals.in.transverse.rows | -0.01827 | -0.35598 | -0.01121 | 0.005514 | 0.145048 | -0.21163 | 0.035356 | -0.0447 | -0.09408 | 0.011963 | -0.08162 | 0.032454 | -0.00885 |
| Lamellae.on.finger.IV | -0.43784 | -0.40551 | -0.13406 | -0.00589 | -0.03758 | 0.058759 | 0.083982 | -0.09748 | -0.01877 | -0.01791 | -0.06474 | 0.02147 | 0.038261 |
| Lamellae.on.toe.IV | 0.377613 | 0.320187 | 0.00662 | 0.003812 | 0.119521 | -0.00361 | -0.10828 | 0.204554 | -0.00576 | 0.057694 | 0.031965 | -0.04064 | 0.011977 |
